# *Crataegus* Species as a Source of Geroprotective Phytochemicals: Biological Activities, Molecular Mechanisms and Therapeutic Perspectives

**DOI:** 10.3390/life16091478

**Published:** 2026-09-04

**Authors:** Assem Kydyrbayeva, Tamara Shalakhmetova, Alina Smalinskiene, Moldir Sharipova, Bulat Aikeshev, Zhuzzhan Kuralai

**Affiliations:** 1 Department of Biophysics, Biomedicine and Neuroscience, Al-Farabi Kazakh National University, Almaty 050040, Kazakhstan; kydyrbaeva.a@kaznmu.kz; 2 Department of Zoology, Histology and Cytology, Al-Farabi Kazakh National University, Almaty 050040, Kazakhstan; 3 Institute of Biological Systems and Genetic Research, Lithuanian University of Health Sciences, 44307 Kaunas, Lithuania; 4 School of Natural Sciences, Astana International University, Astana 010000, Kazakhstan; 5 Department of Molecular Biology and Medical Genetics, S.D. Asfendiyarov Kazakh National Medical University, Almaty 050012, Kazakhstan

**Keywords:** *Crataegus*, phytochemistry, flavonoids, geroprotection, cellular senescence, antioxidants, medicinal plants, SASP, senotherapeutics, inflammaging, proanthocyanidins, Nrf2

## Abstract

Population aging has intensified the search for natural compounds capable of promoting healthy aging and preventing age-related diseases. Among medicinal plants, species of the genus *Crataegus* (hawthorn) have attracted increasing attention because of their rich phytochemical composition and broad spectrum of biological activities. This review summarizes and critically evaluates current evidence on the geroprotective potential of *Crataegus* species, integrating data from phytochemical, pharmacological, and mechanistic studies. Particular attention is given to the major bioactive constituents of *Crataegus*, including flavonoids, oligomeric proanthocyanidins, phenolic acids, and triterpenoids, which exhibit antioxidant, anti-inflammatory, cardioprotective, neuroprotective, metabolic, and anticancer properties. We discuss the growing evidence that these phytochemicals target multiple hallmarks of aging by modulating oxidative stress, chronic low-grade inflammation (inflammaging), mitochondrial dysfunction, cellular senescence, and the senescence-associated secretory phenotype (SASP). Their biological effects are mediated through key signaling pathways involved in cellular homeostasis and longevity, including Nrf2/ARE, NF-κB, PI3K/Akt/mTOR, AMPK, and SIRT1. The review also critically examines findings from in vitro, in vivo, and available clinical studies, highlighting both the therapeutic potential and current limitations of *Crataegus*-based interventions for age-related disorders. Although preclinical evidence strongly supports the multi-target geroprotective properties of *Crataegus* species, clinical validation remains limited. Overall, *Crataegus* represents a promising source of natural geroprotective agents with the potential to promote healthy aging through the modulation of multiple aging-related molecular pathways. Future well-designed clinical studies are essential to establish their efficacy, safety, optimal dosage, and long-term therapeutic value in humans.

## 1. Introduction

According to the World Health Organization (WHO), by 2050 the number of people aged 60 years and older is projected to 2.1 billion, accounting for 22% of the global population [1]. Aging is the primary risk factor for the development of most chronic diseases, including cardiovascular diseases, cancer, metabolic disorders, and neurodegenerative conditions, which together represent the leading causes of morbidity and mortality worldwide [2]. Rather than being an inevitable consequence of time, aging is now recognized as a complex and potentially modifiable biological process driven by interconnected molecular and cellular mechanisms. The updated Hallmarks of Aging framework proposed by López-Otín et al. identifies twelve interconnected hallmarks—including genomic instability, telomere attrition, epigenetic alterations, loss of proteostasis, disabled macroautophagy, chronic inflammation, dysbiosis, and cellular senescence—that collectively contribute to functional decline and age-related diseases [2]. Consequently, contemporary geroscience has shifted its focus from treating individual age-associated diseases toward targeting the fundamental biological mechanisms of aging to extend healthspan and reduce the burden of chronic diseases [2]. Studies indicate that more than half of individuals over the age of 65 suffer from multiple chronic diseases simultaneously, substantially increasing the burden on healthcare systems [3,4,5]. The rapid expansion of the aging population has therefore intensified interest in developing novel geroprotective interventions capable of delaying biological aging and preventing multiple age-related disorders simultaneously [6,7].

The updated Hallmarks of Aging framework provides a comprehensive biological basis for understanding the progressive functional decline associated with aging. Rather than representing independent processes, the twelve hallmarks constitute an interconnected network in which alterations in one hallmark frequently accelerate dysfunction in others. Among these hallmarks, cellular senescence has emerged as a central driver of aging because it interacts closely with genomic instability, mitochondrial dysfunction, chronic inflammation, disabled macroautophagy, and dysregulated intercellular communication. Owing to these extensive interactions, interventions targeting cellular senescence may simultaneously influence multiple hallmarks of aging and therefore represent one of the most promising strategies for extending healthspan and delaying age-related diseases [8,9,10].

One of the key determinants of age-related pathologies and functional decline is cellular senescence [11].

The accumulation of senescent cells is increasingly recognized as a critical driver of tissue dysfunction during aging, contributing to the pathogenesis of multiple diseases [7]. These cells secrete a wide array of bioactive molecules, including pro-inflammatory cytokines, chemokines, growth factors, proteases, lipid mediators, extracellular vesicles, and pro-coagulant proteins, collectively known as the senescence-associated secretory phenotype (SASP). These bioactive mediators promote chronic inflammation and tissue dysfunction [8]. Even a small number of senescent cells can markedly impair the function of organs and physiological systems [12].

Currently, cellular senescence is considered one of the most tractable and therapeutically promising mechanisms underlying aging and age-associated diseases [13,14]. A growing body of evidence indicates that the elimination of senescent cells is highly beneficial [15]. Targeted clearance of senescent cells confers substantial advantages by alleviating numerous pathological conditions, including steatosis, atherosclerosis, atrophy, cardiomyocyte hypertrophy, cataracts, renal glomerulosclerosis, sarcopenia, oncogenesis, cancer recurrence, osteoarthritis, and neurodegenerative diseases [16,17,18,19,20]. Since senescence is deeply interconnected with other hallmarks of aging, targeting this mechanism can indirectly influence a large number of—if not all—other hallmarks [9].

The modern concept of geroscience is based on the idea that targeting the fundamental biological mechanisms of aging can simultaneously slow the progression of several age-related diseases and increase healthspan. In this regard, compounds with a multi-target mechanism of action that can simultaneously modulate several signs of aging are of particular interest. Among such compounds, natural phytochemicals are being the subject of growing interest due to their ability to influence key signaling pathways that regulate cellular homeostasis, inflammation, oxidative stress, and cellular senescence.

These insights have stimulated extensive efforts to identify pharmacological agents capable of eliminating senescent cells in humans [21,22,23]. Rising evidence promotes the effectiveness of geroprotective interventions, particularly senotherapeutic agents, which comprise senolytics and senomorphics [9].

Among these, specific focus has been drawn to medicinal plants such as the genus *Crataegus*, which possesses notable therapeutic and anti-aging potential. *Crataegus* species have long been used in traditional medicine for the treatment of various disorders, including cardiovascular and inflammatory conditions, immune system dysfunctions, as well as liver and kidney pathologies. Their bioactive constituents—including polyphenols, flavonoids (e.g., hyperoside, rutin, and vitexin; approximately 0.1–1% in fruits and 1–2% in leaves and flowers), oligomeric proanthocyanidins (1–3%), and triterpenic acids (0.5–1.4% in fruits; see Section 3.2 for a detailed phytochemical profile)—exert antioxidant, anti-inflammatory, and cardioprotective effects [24,25] and play a key role in the rejuvenating properties attributed to these plants [26].

Traditional herbal remedies have gained particular relevance as potential candidates for the prevention and even treatment of chronic diseases and age-related conditions [27]. According to WHO estimates, nearly two-thirds of the global population employs medicinal plants for managing health conditions owing to their accessibility, safety, and effectiveness [28]. Modern research further supports the pharmacological value of these plants in the prevention and therapy of chronic diseases [29]. Advances in phytochemistry and biotechnology have opened new opportunities for investigating the geroprotective potential of *Crataegus* species within the framework of anti-aging interventions [30].

The genus *Crataegus* is of particular interest among medicinal plants because of its high content of polyphenolic compounds, broad spectrum of pharmacological activity, and centuries-long history of use in traditional and folk medicine. Accumulated experimental data indicate that the biologically active compounds of *Crataegus* species are capable of simultaneously influencing several key mechanisms of aging, including oxidative stress, chronic inflammation, mitochondrial dysfunction, cellular senescence, and impaired autophagy. This multi-target nature of their action makes species of this genus a promising source of natural geroprotective compounds. At the same time, despite a significant number of studies on the phytochemical composition and pharmacological properties of various *Crataegus* species, their geroprotective potential and molecular mechanisms of action remain insufficiently systematized to date. There is a lack of a comprehensive analysis that synthesizes current data on the effects of *Crataegus’s* bioactive compounds on key markers of aging, cellular senescence, and related signaling pathways.

In light of this, this review aims to critically summarize and analyze current data on the phytochemical composition, biological activity, and molecular mechanisms of action of species of the genus *Crataegus*, in addition to evaluation of their efficacy as natural geroprotective agents for the prevention of age-related diseases and the promotion of healthy aging. Specific attention is paid to the effects of biologically active compounds from *Crataegus* on key signs of aging, senotherapy effects, and the regulation of signaling pathways involved in maintaining cellular homeostasis and longevity processes.

## 2. Review Methodology

A structured literature search was conducted utilizing electronic databases such as PubMed, Scopus, and Web of Science Core Collection to identify publications related to the botanical characteristics, phytochemical composition, biological activities, pharmacological properties, molecular mechanisms, and geroprotective potential of *Crataegus* species. The search covered studies published between January 2014 and March 2026, while earlier landmark publications were additionally included when they provided the conceptual basis for aging biology, cellular senescence, or the pharmacology of hawthorn.

The search strategy combined Medical Subject Headings (MeSH), where applicable, and free-text keywords using the Boolean operators AND and OR. The primary search terms included “*Crataegus*”, “hawthorn”, “botanical characteristics”, “phytochemical composition”, “biological activity”, “pharmacological potential”, “flavonoids”, “polyphenols”, “proanthocyanidins”, “phenolic acids”, “triterpenoids”, “aging” OR “ageing”, “healthy aging”, “geroprotector*”, “cellular senescence”, “SASP”, “senotherapeutics”, “inflammaging”, “oxidative stress”, “Nrf2”, “NF-κB”, “PI3K/Akt/mTOR”, “AMPK”, and “SIRT1”.

Only peer-reviewed articles published in English were considered. Eligible publications involved original research articles, systematic reviews, narrative reviews, and clinical studies investigating the botanical characteristics, phytochemical composition, biological activities, pharmacological properties, molecular mechanisms, or geroprotective effects of *Crataegus* species and their bioactive constituents. Conference abstracts without full-text availability, editorials, letters, duplicate publications, non-peer-reviewed sources, and studies unrelated to *Crataegus* or aging-associated mechanisms were excluded.

The retrieved records were screened by title and abstract, followed by full-text evaluation of potentially relevant studies. Reference lists of the selected articles were also manually examined to identify additional eligible publications.

As this article represents a narrative review, the objective was to provide a comprehensive and critical synthesis of the available evidence rather than to perform a systematic review or meta-analysis. Therefore, no formal PRISMA selection process or quantitative assessment of publication bias was performed.

## 3. Botanical and Phytochemical Background of *Crataegus*

### 3.1. Botanical Characteristics and Distribution of Crataegus

Hawthorn is one of the oldest and most widely used medicinal plants. *Crataegus* L. is a genus in the subfamily Maloideae of the family Rosaceae, widely distributed across Europe and Asia [31]. A distinctive feature of *Crataegus* is its remarkable ecological plasticity, which enables it to thrive in diverse climatic conditions and across different altitudes [32]. The genus is widely spread throughout the temperate regions of Europe, North America, North Africa, India, China, and Western Asia. Certain species are characteristic of specific geographic regions: *Crataegus pinnatifida* Bunge is known as the Chinese hawthorn, *C. pubescens* Steud. as the Mexican hawthorn, and *C. cuneata* Rehder ex C.K. Schneid as the Japanese hawthorn. In Europe, *C. laevigata* (Poir.) DC and *C. monogyna* Jacq. are common; in the Middle East, *C. oxyacantha* L. and *C. aronia* var. *aronia* (L.) Bosc. ex DC are found; in North America, *C. phaenopyrum* Borkh.; and in Russia, *C. ambigua* C.A. Mey. ex A.K. Becker [33,34,35,36].

**Figure 1:**
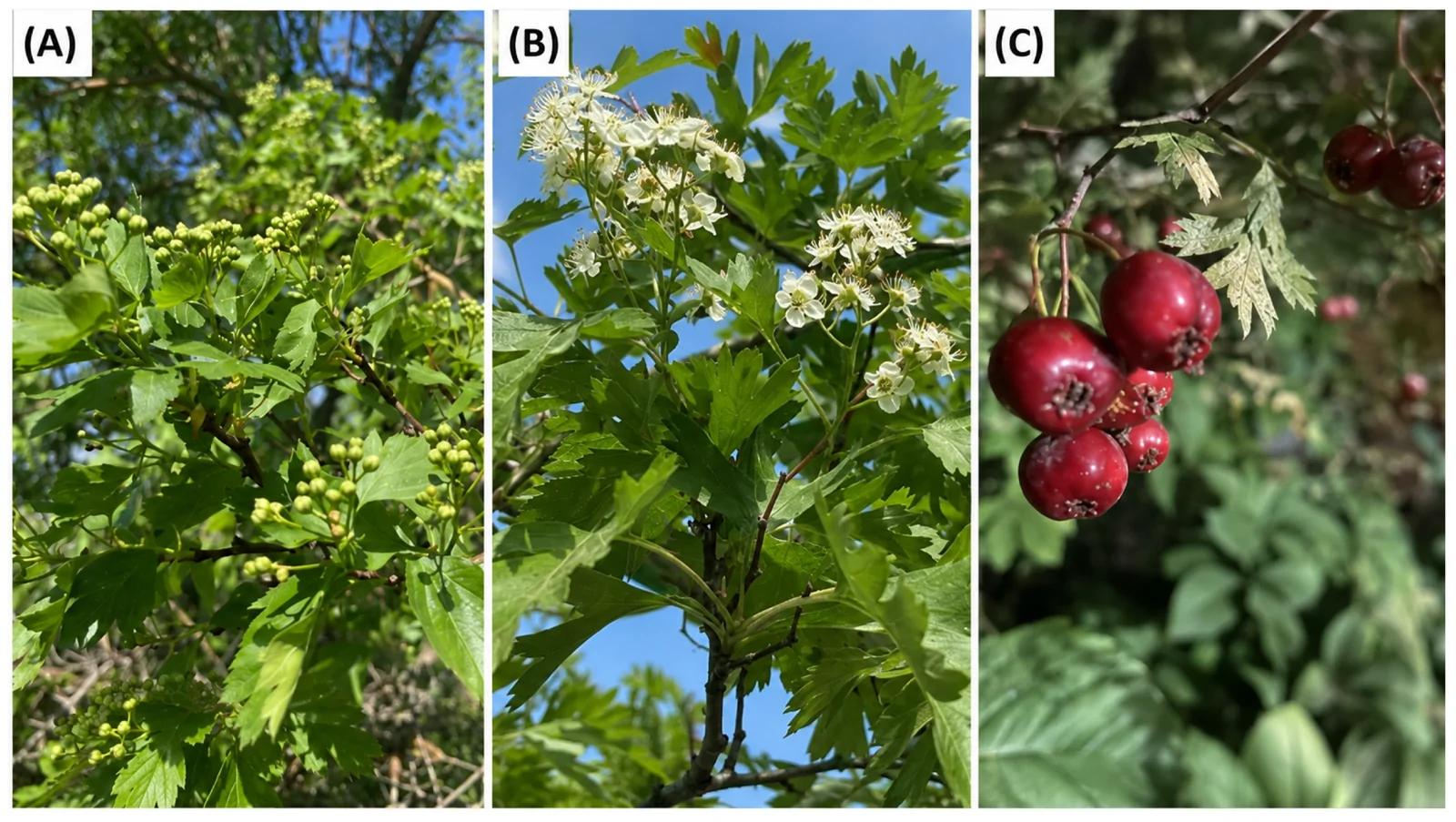
Botanical characteristics of *Crataegus almaatensis* Pojark. Photographs show the typical phenological stages of the species: (**A**) vegetative shoot with developing inflorescences and characteristic deeply lobed leaves; (**B**) flowering branch with white, five-petaled actinomorphic flowers arranged in corymbose inflorescences; (**C**) mature bright-red pomaceous fruits (haws) with smooth glossy surface. The images illustrate the main diagnostic morphological features of *C. almaatensis*, including leaf shape, inflorescence structure, and fruit morphology.

In Kazakhstan, the genus is represented by a wide diversity of species well-known for its high ecological adaptability. According to the Flora of Kazakhstan [37], seven species are recorded in the country: *C. sanguinea* Pall., *C. altaica* Lge., *C. pontica* C. Koch., *C. songarica* C. Koch., *C. turkestanica* Pojark., *C. almaatensis* Pojark., and *C. ambigua* C.A. Mey. ex A.K. Becker [38,39]. Among these, *Crataegus almaatensis* Pojark. is endemic to the mountainous region of the Ile Alatau and represents a unique species with a restricted geographic range (Figure 1).

This species forms compact trees 3–4 m tall with a dense crown and characteristic morphological traits, with its natural distribution limited to the surroundings of Almaty. The leaves are oval, dark green on the upper surface and lighter underneath, deeply divided into 5–7 lobes, and covered with trichomes—a key diagnostic feature of the species. The flowers are arranged in loose corymbose inflorescences with a whitish-yellow corolla up to 18 mm in diameter, twenty stamens with purple anthers, and an ovary composed of 3–5 carpels. The fruits are broadly ovoid, dark violet in color, with juicy reddish pulp and containing 3–5 seeds, allowing reliable distinction of this endemic species from other members of the genus *Crataegus* [40].

Thus, hawthorn species of the genus *Crataegus* represent a highly adaptable and taxonomically diverse group of plants with a broad geographic distribution. Among them, particular importance is attributed to endemic species such as *Crataegus almaatensis*, which possess high potential and accessibility for the development of medicinal plant-based therapeutics.

### 3.2. Phytochemical Composition of Crataegus

More than 150 chemical constituents have been identified across different *Crataegus* species [41]. The fruits, leaves, and flowers of hawthorn contain a wide variety of phytochemicals. In particular, flavonoids are present in varying concentrations—ranging from 0.1% to 1% in fruits and from 1% to 2% in leaves and flowers—and include hyperoside, rutin, vitexin, acetylvitexin-2″-O-rhamnoside, and vitexin-2″-O-rhamnoside [42,43,44]. Oligomeric proanthocyanidins (OPCs), found in fruits and leaf–flower mixtures at concentrations of 1–3%, consist of chains of flavan-3-ol units. In addition, the plant contains organic acids (2–6%), triterpenic acids (0.5–1.4% in fruits, including oleanolic and ursolic acids), phenolic acids (chlorogenic and caffeic acids), saponins, as well as amines and sterols [44,45,46]. Among these, flavonoid glycosides and oligomeric proanthocyanidins are considered the primary compounds responsible for the genus’s biological activity [47].

**Figure 2:**
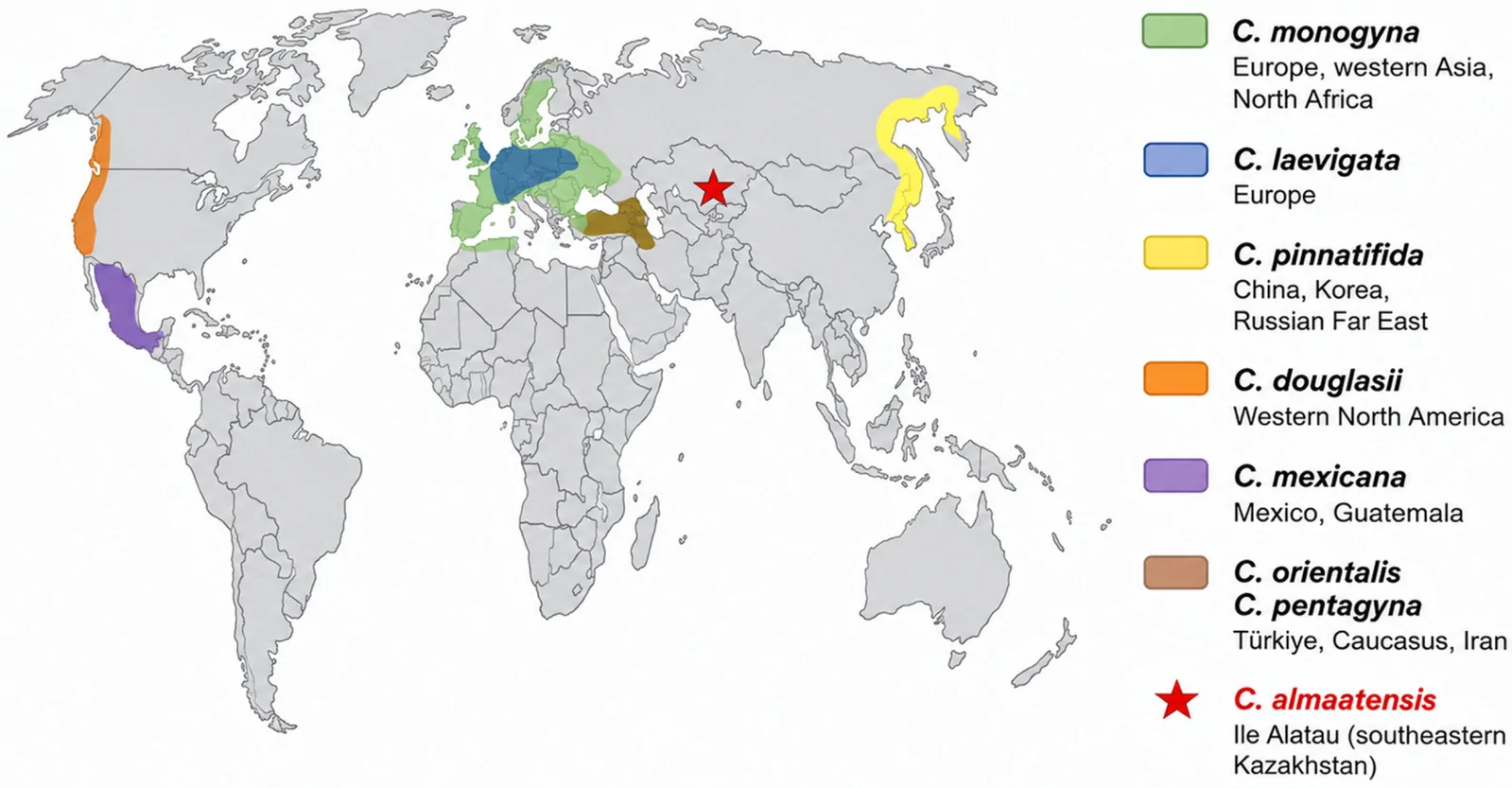
Approximate native distribution of representative *Crataegus* species discussed in this review. Distribution areas are schematic and were compiled by the authors based on published floristic and taxonomic sources.

It is noteworthy to consider the geographic distribution of *Crataegus* species, as environmental conditions can influence their phytochemical composition and, consequently, their pharmacological activity. Such differences in the levels of bioactive compounds directly affect the therapeutic potential of various *Crataegus* species [48]. Studies of *C. pentagyna* further demonstrate substantial phytochemical diversity among different plant organs, including leaves, flowers, fruits, and roots, with variations in phenolic and flavonoid composition accompanied by antioxidant and antimicrobial activities [49,50]. Table 1 presents the phytochemical profiles of selected *Crataegus* species, and Figure 2 illustrates key compounds with proposed geroprotective activity.

The typical phytochemical profile of hawthorn is generally consistent across species and plant organs: flavonoids (rutin, hyperoside, and vitexin C-glycosides), flavan-3-ols ((−)-epicatechin), and phenolic acids (chlorogenic acid) predominate, although their proportions vary between leaves, flowers, and fruits [40,51]. The highest phenolic content has been reported in the leaves of the Kazakh endemic *C. almaatensis*, which exhibit the greatest total phenolic content (TPC) and the strongest radical-scavenging activity, whereas the fruits contain comparatively lower levels (Bekbolatova et al.) [40]. Seasonality and fruit maturity are critical factors: in *C. pentagyna*, the peak concentration of (−)-epicatechin in fruits occurs in August, which is essential for raw-material standardization [51]. Importantly, Ornelas-Lim et al. developed a standardized extract of *C. mexicana* leaves based on (−)-epicatechin and rutin using HPLC-DAD validated according to ICH/Eurachem guidelines; the methanolic extract demonstrated pronounced vasodilatory activity ex vivo [52].

Inter-species and geographical differences confirm the high chemotypic variability of the genus; therefore, data must be compared strictly by plant organ, solvent, and season. The optimal analytical framework includes TPC/TFC screening, LC-MS or HPLC-DAD profiling, and validation of selected marker compounds for each specific species [40,53].

**Table 1:** Phytochemical Composition of Different Plant Organs of the Genus *Crataegus*.

Species	Plant Part	Major Compounds (Key Data)	Analytical Method	Biological Activity	Ref.
*C. monogyna*	Leaves, flowers (Algeria)	Catechin, rutin, tannins (TPC 1.65 mg GAE/g)	HPLC-UV/Vis; DPPH; molecular docking	Antioxidant; α-amylase inhibition; non-toxic	[52]
*C. monogyna*	Flowers, fruits (Mediterranean)	Phenolics, flavonoids, tocopherols, ascorbic acid	HPLC-RI; GC-FID; spectrophotometry	High antioxidant activity; flowers richest in tocopherols	[53]
*C. pinnatifida*	Fruits (Asia)	>50 flavonoids; total phenolics (2–249 mg/g)	RT-PCR; skin analysis	Improved skin hydration and wrinkle reduction	[54]
*C. pinnatifida*	Fruit pulp and peel (China)	Phenolic acids, flavonoids, proanthocyanidins, triterpenoids	LC-MS/MS	Antioxidant; anti-inflammatory; cardioprotective	[30]
*C. azarolus*	Leaves (Algeria)	Catechin, hyperoside (TPC 396 mg GAE/g)	TLC; NMR; MS; Folin–Ciocalteu	Antioxidant; antihypertensive	[55]
*C. orientalis*	Fruits (Türkiye)	Chlorogenic acid, hyperoside, quercetin	LC-HRMS; DPPH; FRAP	Antioxidant	[56,57]
*C. mexicana*	Leaves (Mexico)	Epicatechin, rutin (TPC 190 mg GAE/g)	Validated HPLC-DAD	Vasorelaxant activity (Emax ≈ 100%)	[50]
*C. pentagyna*	Leaves, flowers, fruits (Romania)	Chlorogenic acid, epicatechin, proanthocyanidins	LC-MS/MS metabolite profiling; ex vivo vascular assay	Endothelium-dependent vasorelaxation; arginase I inhibition	[51]
*C. almaatensis*	Leaves, flowers, fruits (Kazakhstan)	Hyperoside, rutin, chlorogenic acid, cyanidin-3-glucoside	LC-HRMS/MS; DPPH; Folin–Ciocalteu	Highest polyphenol content in leaves (TPC 218 mg/g)	[58]

**Figure 3:**
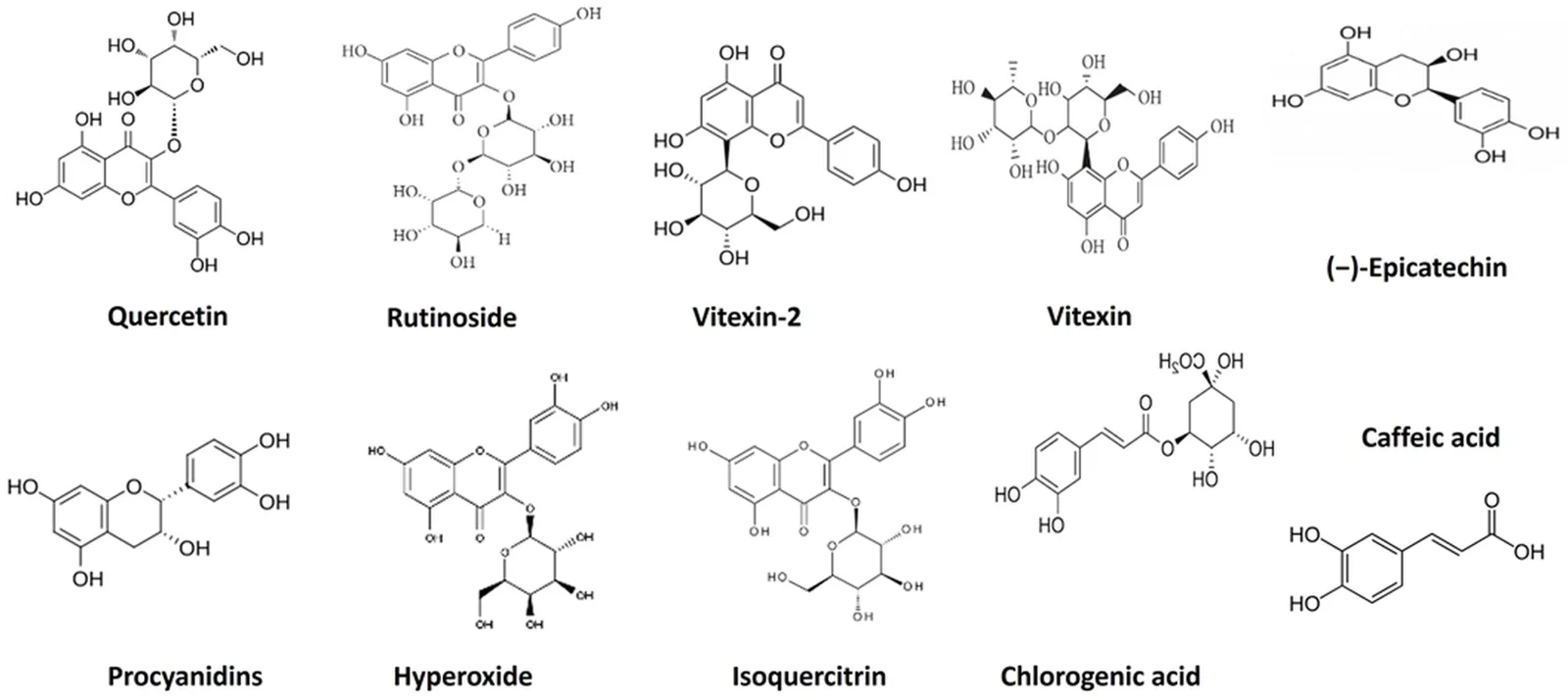
Structures of key *Crataegus* compounds with proposed geroprotective potential.

Comparing across the ten datasets summarized in Table 1, several patterns emerge. Hyperoside and rutin are the most consistently reported marker flavonoids, present in leaves of *C. monogyna*, *C. azarolus*, *C. douglasii*, *C. mexicana*, *C. pentagyna*, and *C. almaatensis*, whereas (−)-epicatechin and procyanidins predominate in fruit-derived preparations (*C. pinnatifida*, *C. mexicana*, *C. pentagyna*). The chemical structures of these and other major phenolic constituents identified in *Crataegus* species are presented in Figure 3. Where multiple organs were compared directly within the same species, leaves consistently showed higher total phenolic content than fruits (e.g., *C. almaatensis* leaves, 218 mg/g, versus fruits, 88–92 mg/g), suggesting that leaf material may generally be a more concentrated raw material for phenolic-driven bioactivity. However, this comparison is confounded by differences in extraction solvents and quantification methods across studies. Vasodilatory activity has so far been demonstrated only for validated leaf extracts of *C. mexicana* and *C. pentagyna* using an essentially identical HPLC-DAD/ICH-Eurachem protocol from the same research group; therefore, this specific pharmacological endpoint cannot yet be generalized across the wider genus. Overall, the methodological standardization applied to *C. mexicana* and *C. pentagyna* is the exception rather than the norm among the studies summarized in Table 1, limiting direct cross-species and cross-organ comparisons of phytochemical potency.

## 4. Pharmacological Properties of *Crataegus*

Recent findings on *Crataegus* convincingly demonstrate a broadening spectrum of its pharmacological activities—from pronounced antioxidant and anti-inflammatory effects to cardio- and neuroprotective actions—largely attributable to the plant’s rich polyphenolic composition. These data clearly highlight the multifaceted pharmacological potential of *Crataegus*, supporting its effectiveness in the prevention and adjunctive treatment of various pathological conditions in line with its well-characterized biochemical profile. In particular, antioxidant constituents such as flavonoids and oligomeric procyanidins represent the major bioactive components responsible for its beneficial effects on the cardiovascular system, including blood-pressure reduction, improvement of coronary and peripheral circulation, and protection against ischemic injury. The strong antioxidant properties of *Crataegus* extracts, associated with their phenolic constituents, have been demonstrated in recent studies [59]. These antioxidant effects may contribute to the attenuation of oxidative stress through reactive oxygen species scavenging and inhibition of lipid peroxidation, thereby limiting cellular and tissue damage associated with various chronic diseases, including cardiovascular and neurodegenerative disorders [44,54,60,61,62].

### 4.1. Antioxidant Mechanisms of Crataegus

The antioxidant potential of *Crataegus* is driven by its abundant pool of phenolic compounds, including flavonoids (hyperoside, rutin, quercetin, vitexin, and others), proanthocyanidins, and phenolic acids. A procyanidin-rich phenolic profile with corresponding antioxidant activity has been identified in *C. monogyna* bark extracts using low-pressure liquid chromatography fractionation [63]. This phytochemical profile has also been consistently demonstrated in the leaves and flowers of various *Crataegus* species by Alirezalu et al. [64] and further confirmed in the berries of *C. monogyna* by Cristea et al. [65].

Antioxidant activity varies depending on species, plant organ, extraction method, and storage conditions. The wide ranges of total phenols (7.21–87.73 mg GAE/g), flavonoids (2.27–17.40 mg rutin eq./g), and reducing capacity (up to 4.65 mmol Fe^2+^/g via FRAP) reported across leaves and flowers of 14 *Crataegus* species further highlight a strong species- and organ-specific antioxidant response [64]. Similarly, alcoholic extracts of *C. monogyna* fruits exhibit strong radical-scavenging capacity in DPPH• and ABTS•^+^ assays, along with marked reducing power, with HPLC analyses identifying (−)-epicatechin, catechin, and procyanidins B1/B2 as the major constituents [65].

Multiple studies demonstrate that *Crataegus* extracts exert potent free radical-scavenging activity in vitro in models of chemically induced oxidative stress. Antioxidant activity assessed using DPPH• and ABTS•+ assays reveals the ability of extracts to neutralize radicals through electron or proton transfer. Aqueous and alcoholic extracts of *C. monogyna* efficiently quench free radicals, exhibit high reducing ability, and demonstrate Fe^2+^-chelating capacity; notably, floral aqueous extracts contain higher flavonoid levels than leaf extracts [66].

Cell-based studies by Quynh T. N. Nguyen et al. [67] showed that berry extracts of *C. laevigata* reduce ROS levels and suppress key pro-inflammatory mediators, including MAPKs/AP-1, NF-κB, and NFAT, consistent with their antioxidant activity and DPPH/ABTS results. In vivo data further validate the translational relevance of *Crataegus* antioxidant and anti-inflammatory effects. In a CCl_4_-induced liver fibrosis model in rats, Alaaeldin A. Hamza et al. [68] demonstrated that polyphenolic extracts of *Crataegus oxyacantha* decrease malondialdehyde (MDA) and protein carbonyls, enhance superoxide dismutase (SOD) activity, reduce collagen deposition and the expression of α-SMA, TGF-β1, NF-κB, and pro-inflammatory cytokines (IL-1β, TNF-α), and restore liver histoarchitecture.

In a pesticide-induced neurotoxicity model, Mongi Saoudi et al. [69] reported that aqueous extracts of *C. oxyacantha* reduce lipid peroxidation in brain tissue, increase antioxidant enzyme expression (SOD, CAT, GPx), and decrease DNA fragmentation, demonstrating a characteristic antioxidant–neuroprotective profile. These antioxidant and neuroprotective effects are consistent with the broader pharmacological properties reported for *Crataegus* species [70]. In food-technology applications, Natalia Żurek, Michał Świeca, and Ireneusz T. Kapusta [48] produced nutraceutical powder SPE from berries, leaves, and flowers of six *Crataegus* species containing very high phenolic concentrations (31.50–66.06 mg/g), with maximum activity in fruit fractions (quenching O_2_•^−^ and OH•), underscoring the applied potential of standardized phenolic concentrates. Clinically and pathophysiologically, the review by Eujin Kim, Eungyeong Jang, and Jang-Hoon Lee [71] summarizes that *Crataegus* antioxidant mechanisms are relevant to a broad range of hepatic disorders (toxic, alcoholic, NAFLD, HCC), where oxidative stress is a central pathogenic node.

Additionally, vinegar produced from *Crataegus tanacetifolia* has been shown to possess strong antioxidant activity. In vitro assays demonstrated DPPH• inhibition of 73.8–80.1% and ABTS•+ inhibition of 78.6–82.5%, along with Fe^2+^-chelating capacity of 31–33% [72]. These findings indicate that even fermented *Crataegus* products retain high phenolic content (328–467 mg GAE/mL) and may serve as dietary antioxidant sources.

Finally, for phytopharmaceutical standardization, Christian Ornelas-Lim et al. [50] emphasize the importance of selecting appropriate chemical markers (e.g., epicatechin and rutin) to ensure reproducible antioxidant activity in *Crataegus* extracts.

Collectively, the studies by Alirezalu et al. (2018) [64], Cristea et al. (2024) [65], Keser et al. (2014) [66], Nguyen et al. (2021) [67], Hamza et al. (2020) [68], Saoudi et al. (2019) [69], Kim, Jang and Lee (2022) [71], and Żurek, Świeca and Kapusta (2024) [72] demonstrate that the antioxidant potential of *Crataegus* is determined by its phenolic composition, enhanced by appropriate extraction methods and conditions, and consistently manifested from chemical assays to cellular and animal models, highlighting hawthorn as a promising geroprotective candidate.

Beyond its antioxidant properties, the pharmacological potential of *Crataegus* also extends to vascular effects. In particular, a vasorelaxant effect has been reported for flower extracts of the Mexican species *C. gracilior*, further supporting the vasoprotective potential of *Crataegus* across different species [73].

It is crucial to recognize that the available evidence is derived predominantly from chemical antioxidant assays and in vitro studies. Only a limited number of investigations involving WS^®^ 1442, hawthorn vinegar, and nutraceutical powder preparations have evaluated complex whole-extract formulations, while antioxidant biomarkers have not yet been adequately assessed in human clinical samples. Considerable variability is also evident across *Crataegus* species, plant organs, extraction procedures, and analytical methods, with reported total phenolic contents ranging from 7.21 to 87.73 mg GAE/g. Accordingly, the antioxidant potential of *Crataegus* should be interpreted in the context of this substantial methodological and phytochemical heterogeneity rather than regarded as a uniform, species-independent property of the genus.

### 4.2. Anti-Inflammatory Activity

Polyphenols and triterpenoids from *Crataegus* species exhibit pronounced anti-inflammatory properties, acting through multiple interconnected signaling pathways of the innate and adaptive immune systems. The major bioactive constituents—flavonoids (hyperoside, quercetin, vitexin, rutin), proanthocyanidins, and triterpenic acids (ursolic and oleanolic acids)—exert combined antioxidant and anti-inflammatory effects by suppressing excessive production of reactive oxygen species (ROS), cytokines, and inflammatory mediators [67,74,75].

Quynh T. N. Nguyen et al. [67], using an LPS-stimulated human keratinocyte (HaCaT) model, demonstrated that a 70% ethanol extract of *Crataegus laevigata* berries markedly reduced ROS levels and inhibited activation of key inflammatory signaling pathways -MAPK/AP-1, NF-κB, and NFAT. This was accompanied by decreased transcription of the pro-inflammatory chemokines TARC/CCL17, MDC/CCL22, RANTES/CCL5, and IL-8, confirming the direct anti-inflammatory and antioxidant effects of *Crataegus* at the cellular level. These findings underscore the ability of *Crataegus* extracts to modulate oxidative–inflammatory responses at early stages of cell activation.

Triterpenoids isolated from *C. pinnatifida* leaves exhibit strong anti-inflammatory activity by reducing nitric oxide (NO) production and suppressing the expression of iNOS, COX-2, TNF-α, IL-1β, and IL-6 genes in RAW 264.7 macrophages. Molecular docking, enzymatic assays, and in vitro models confirmed that these compounds directly interact with SRC tyrosine kinase, inhibiting its phosphorylation and subsequently blocking STAT3-dependent inflammatory signaling [75].

Similar findings have been reported for ursolic acid, which, according to several studies, inhibits NF-κB nuclear translocation and decreases COX-2 and iNOS expression, thereby reducing pro-inflammatory cytokine production and limiting apoptosis of inflamed cells [76,77].

In animal models, Amrati et al. [45] demonstrated that a hydroethanolic extract of *C. monogyna* leaves and stems dose-dependently suppressed carrageenan-induced paw edema in rats, achieving near-complete anti-edematous effect (~99% at 6 h at a dose of 1000 mg/kg) and providing hepatoprotective activity in a model of acetaminophen-induced hepatotoxicity. These findings support the potent anti-inflammatory action of the extract, as inhibition of carrageenan-induced edema is a widely accepted in vivo model for assessing anti-exudative and antiflogistic activity.

Additionally, Żurek et al. [48] comprehensively characterized the polyphenolic composition of berries, leaves, and flowers from six *Crataegus* species. Their metabolomic analysis demonstrated that these plant organs are rich in flavan-3-ols, procyanidins, phenolic acids, and flavonoids, supporting the well-established antioxidant and anti-inflammatory potential of *Crataegus*. These extracts not only inhibited lipoxygenase (LOX) activity—reducing leukotriene synthesis—but also suppressed cyclooxygenase-2 (COX-2) and xanthine oxidase (XO), making *Crataegus* seeds a promising source of multitarget anti-inflammatory agents. Moreover, principal component analysis (PCA) demonstrated a strong association between flavan-3-ol content and LOX/COX-2 inhibitory activity, suggesting that these compounds may contribute to the observed anti-inflammatory effects. However, PCA is a correlational analytical approach and does not establish a causal relationship.

Collectively, in vitro and in vivo findings confirm that *Crataegus* species possess robust and multifaceted anti-inflammatory potential. Phenolic compounds (flavonoids, proanthocyanidins) and triterpenoids play a central role by modulating key inflammatory signaling pathways—NF-κB, MAPK, STAT3, COX-2, and iNOS—thereby reducing the expression of pro-inflammatory mediators (TNF-α, IL-1β, IL-6, NO). In cellular models, *Crataegus* extracts suppress cytokines and chemokines, while in animal models, they reduce carrageenan-induced edema, provide hepatoprotection, and attenuate oxidative-inflammatory tissue damage. Additionally, procyanidins from *Crataegus* seeds inhibit LOX, COX-2, and XO, confirming their role in regulating eicosanoid and free radical pathways associated with inflammation.

Overall, the available evidence for the anti-inflammatory effects of *Crataegus* is based predominantly on in vitro studies, including HaCaT keratinocytes and RAW264.7 macrophages, together with a limited number of short-term rodent models of experimentally induced inflammation. Although these studies provide mechanistic support for the modulation of inflammatory pathways, including NF-κB, COX-2, and LOX, direct clinical evidence remains limited, particularly with respect to inflammatory biomarkers in humans. Moreover, the effective concentrations reported in vitro may not necessarily reflect the systemic exposure achievable after oral administration of *Crataegus* extracts. These limitations highlight the need for well-designed pharmacokinetic and clinical studies before the observed mechanistic effects can be reliably translated into therapeutic anti-inflammatory applications.

### 4.3. Cardioprotective Effects of Crataegus

The cardioprotective activity of *Crataegus* is primarily attributed to its polyphenolic profile (flavonoids and oligomeric proanthocyanidins), which provides a combination of antioxidant, vasoprotective, anti-inflammatory, and antiarrhythmic effects [78]. At the level of the myocardium and vascular endothelium, such extracts exhibit a positive inotropic effect independent of cAMP stimulation, prolongation of the action potential and refractory period, enhancement of NO synthesis and endothelial protection, as well as attenuation of ischemia–reperfusion injury and hypertensive hypertrophy [79,80]. This vasoprotective, NO-dependent activity is not confined to a single species: a comparable vasorelaxant effect has been reported for flower extracts of the Mexican species *C. gracilior* [73], reinforcing the reproducibility of this pharmacological endpoint across geographically distinct *Crataegus* species, although—as noted in Section 3.2—this remains an emerging cross-species pattern rather than a formally demonstrated genus-wide property.

Recent clinical and epidemiological data have drawn particular attention to the standardized leaf-and-flower extract WS^®^1442. In a large retrospective “real-world” cohort study based on the IQVIA database (*n* = 9100; 5-year follow-up), the use of WS^®^1442 was associated with significantly lower cumulative rates of atrial fibrillation/flutter, tachycardia, and other arrhythmias compared with magnesium-potassium therapy (HR for AF = 0.71; 95% CI: 0.64–0.80) [81]. These findings are consistent with experimental observations of action-potential prolongation and the strong antiarrhythmic properties of WS^®^1442.

Mechanistically, the cardioprotective effects of *Crataegus* are multifactorial. A network-pharmacology analysis of *Crataegus* leaves for coronary artery disease identified quercetin, isorhamnetin, and kaempferol as key constituents and highlighted the involvement of PTGS2/COX-2, EGFR, MMP2/9, and pro-inflammatory cytokines, indicating integrated immuno-inflammatory and endothelial modulation [82]. Similar integrated LC-MS-based metabolite profiling combined with network pharmacology has recently been used to decipher the multi-target pharmacological basis of other medicinal plant materials, such as Eucommiae folium in rheumatoid arthritis, and represents a methodological model that could be applied to further validate and refine the specific molecular targets underlying the cardioprotective network of *Crataegus* [83].

Additionally, WS^®^1442 has been shown to stimulate cardiomyogenesis from embryonic stem cells and enhance angiogenesis from cardiac progenitor cells; this activity is partially attributed to its OPC fraction (degree of polymerization DP3–6) and is associated with inhibition of TGFβ/BMP and FGF signaling, as well as upregulation of BDNF and retinoic acid signaling [84]. These findings suggest a potential regenerative role for WS^®^1442 in cardiac repair; however, the in vivo relevance of these effects following myocardial infarction remains to be established.

From the perspective of cardiometabolic burden and post-ischemic remodeling, a review by Olabiyi and de Castro Brás [85] emphasized the central roles of chronic inflammation, oxidative stress, and extracellular-matrix dysfunction in cardiovascular remodeling. Against this mechanistic background, the antioxidant and anti-inflammatory properties of polyphenol-rich preparations such as *Crataegus* provide a rationale for further investigation of their potential role in strategies aimed at limiting adverse left-ventricular remodeling [78,79,84,85].

Moreover, a meta-analysis of randomized placebo-controlled trials in hypertensive patients demonstrated a clinically relevant reduction in systolic blood pressure (approximately −6.6 mmHg after 2–6 months of therapy), further supporting the antihypertensive contribution to the cardioprotective profile of *Crataegus* [86].

Experimental in vivo studies in rat myocardial ischemia–reperfusion models have shown that standardized *Crataegus extract* WS^®^1442 reduces the incidence of ventricular arrhythmias, decreases infarct size and mortality, and attenuates ST-segment elevation. These cardioprotective effects have been associated with antioxidant activity and enhanced endothelial nitric oxide (NO) synthesis [87].

Earlier comprehensive pharmacological reviews have further documented the cardiovascular actions of *Crataegus*, including antioxidant and anti-inflammatory effects, protection of vascular endothelial function, promotion of endothelial relaxation, regulation of lipid metabolism, and antiplatelet activity [88].

Safety and drug-interaction profiles remain critical considerations for clinical use of *Crataegus* preparations. According to a benefit–risk assessment of WS^®^1442 [81], the standardized extract demonstrates good tolerability in patients with chronic heart failure (NYHA II–III), shows no clinically significant drug–drug interactions when used alongside standard therapy, and exhibits a favorable benefit–risk ratio.

However, data from an experimental study in rats [89] indicated that hawthorn flower and leaf extract, alongside its antioxidant effects, can influence the hemostatic system, including increased antithrombin III levels and reduced factor X activity. Although these findings were obtained in an animal model and cannot be directly extrapolated to humans, they suggest that potential interactions with anticoagulant or antiplatelet therapy warrant further investigation.

Collectively, recent evidence demonstrates that the cardioprotective effect of *Crataegus* is mediated through multiple mechanisms—antiarrhythmic actions, vasoprotection, anti-inflammatory modulation, and influence on myocardial regenerative capacity. Integrated experimental, clinical, and pharmacological findings indicate that *Crataegus* exerts multilevel cardioprotection through antioxidant, anti-inflammatory, antiarrhythmic, vasoprotective, and regenerative pathways. Combined with its favorable safety profile, *Crataegus* preparations represent promising adjuncts in cardiovascular therapy and in the prevention of pathological myocardial remodeling.

Of the mechanisms discussed in this section, only those associated with the standardized extract WS^®^1442 are supported by clinical-grade evidence, including randomized controlled trials in chronic heart failure and a large retrospective cohort study (n = 9100) on arrhythmia outcomes. The remaining cardioprotective mechanisms—cardiomyogenesis, OPC-mediated angiogenesis, ischemia–reperfusion protection—remain confined to animal and in vitro models. Because WS^®^1442 is a specific, standardized preparation rather than a generic *Crataegus* extract, its clinical findings should not be assumed to apply to other *Crataegus* species or unstandardized preparations without direct supporting data.

### 4.4. Neuroprotective Properties

The phenolic constituents of *Crataegus*, particularly flavonoids and other polyphenolic compounds, contribute to its neuroprotective potential through antioxidant mechanisms and protection against oxidative neuronal injury [90].

Data on molecular targets are complemented by chemical-pharmacological studies of isolated compounds. Ali et al. [91] isolated nine constituents from *C. oxyacantha* (including β-sitosterol-3-O-β-D-glucopyranoside, lupeol, betulin, betulinic and oleanolic acids, and chrysin) and demonstrated robust inhibition of acetylcholinesterase and butyrylcholinesterase (IC50 for AChE as low as 5.22 μM; for BChE 0.55–15.36 μM), with predicted BBB permeability, supporting a cholinergic contribution to neuroprotection in cognitive disorders.

A substantial body of evidence is derived from spinal cord injury (SCI) models, where “total flavonoids from hawthorn leaves” (TFHL) consistently promote neurological recovery. In the study by Zhang et al. [92], repeated TFHL injections (5–20 mg/kg) improved BBB locomotor scores, reduced lesion cavity size and TUNEL-positive cells, and shifted the balance of Bcl-2 family proteins toward anti-apoptotic signaling (↓ Bax, ↓ cleaved caspase-3, ↑ Bcl-2), corresponding to reduced neuronal apoptosis.

Subsequent work by the same group demonstrated that TFHL additionally stimulates autophagy and axonal regeneration of motoneurons (in vivo and in SMN–astrocyte co-culture), accompanied by improved microenvironmental conditions, increased secretion of neurotrophic factors, and restoration of neuronal microstructure; these effects are attributed to activation of autophagic proteins followed by functional recovery [92].

The link between antioxidant defense and neurotrophic signaling is also evident in combined “behavioral therapy + phytopharmacology” protocols. In a trimethyltin (TMT)-induced neurotoxicity model, Keshvari et al. [93] demonstrated that 12 weeks of combined exercise training and oral hawthorn supplementation produced greater neuroprotective effects than either intervention alone. The combined intervention improved spatial learning and memory in the Morris water maze, modulated hippocampal BDNF/Trkβ signaling and AChE activity, reduced β-amyloid burden, and ameliorated neurohistopathological alterations, including eosinophilic neuronal necrosis and astrogliosis [93]. This highlights the synergistic neuroprotective potential of *Crataegus* within multimodal rehabilitation programs.

At the behavioral level, neurocognitive effects have been demonstrated in diabetes-associated models: two-week oral administration of *Crataegus* extract (100–1000 mg/kg) to streptozotocin-induced diabetic rats improved passive avoidance memory and produced dose-dependent changes in metabolic parameters, including reductions in blood glucose and cholesterol at 100–300 mg/kg and increased HDL at 1000 mg/kg [94].

It should be noted that the dose range tested in this study (100–1000 mg/kg) spans a 10-fold interval, and the original report did not establish a clear dose-dependent relationship for the cognitive and metabolic outcomes; whether lower, more clinically translatable doses would retain efficacy remains to be determined.

In toxic brain injury models, *C. oxyacantha* extract exhibits pronounced antioxidant and anti-inflammatory protection. Saoudi et al. [69] demonstrated that pretreatment with an aqueous extract prevented behavioral impairments (open field, elevated plus maze), reduced lipid peroxidation, restored antioxidant enzymes (SOD, CAT, GPx, GSH), decreased DNA fragmentation, and improved histopathological architecture in rats exposed to combined deltamethrin and chlorpyrifos toxicity; the protective effect was comparable to vitamins C + E.

Experimental studies further support the involvement of antioxidant mechanisms in the neuroprotective effects of *Crataegus*. In a pesticide-induced neurotoxicity model, *C. oxyacantha* extract reduced lipid peroxidation and restored endogenous antioxidant defenses, including SOD, CAT, GPx, and GSH, while improving neurobehavioral and histopathological outcomes [69]. Complementary cell-based evidence indicates that phenolic-rich *Crataegus extracts* can also protect neuronal cells against oxidative injury [90].

The convergence of evidence across mechanistic levels—from behavioral improvements in diabetes-associated memory dysfunction [94] and protection against hypoxia-induced oxidative brain injury [95], through cholinesterase inhibition by isolated metabolites [91], to anti-apoptotic and pro-autophagic neuroprotection in SCI [92,93]—indicates that *Crataegus* possesses multifaceted neuroprotective potential.

As in the preceding sections, the neuroprotective evidence summarized here is derived predominantly from rodent models of chemically induced neurotoxicity, spinal cord injury, and diabetes, together with enzyme-inhibition data for isolated compounds rather than whole *Crataegus* extracts. No clinical studies evaluating cognitive or neurodegenerative endpoints after *Crataegus* administration in humans were identified, and the behavioral improvements reported in these animal models should be regarded as hypothesis-generating rather than as direct evidence of neuroprotection in human cognitive or neurodegenerative disease.

**Convergent Mechanisms and Unresolved Questions Across Section 4.2, Section 4.3 and Section 4.4**

Although presented as separate pharmacological domains, the anti-inflammatory, cardioprotective, and neuroprotective effects described in Section 4.2, Section 4.3 and Section 4.4 converge mechanistically to a substantial degree. NF-κB signaling recurs as a central node across all three domains: its inhibition underlies the anti-inflammatory reduction in TNF-α, IL-1β, and IL-6 (Section 4.2), contributes to the suppression of inflammatory mediators accompanying myocardial ischemia–reperfusion injury (Section 4.3), and underlies attenuation of neuroinflammatory cascades in models of spinal cord injury and toxin-induced neurodegeneration (Section 4.4). Oxidative stress reduction is similarly a shared upstream mechanism, evidenced by consistent reports of decreased malondialdehyde and increased superoxide dismutase and catalase activity across the anti-inflammatory, cardioprotective, and neuroprotective literature summarized in Table 2, which consolidates the specific signaling pathways (NF-κB, MAPK, STAT3, COX-2, iNOS, LOX, XO) associated with each compound and model rather than repeating them in full here. Cytokine regulation—particularly TNF-α and IL-6—is likewise reported as a downstream readout across all three domains.

This mechanistic overlap suggests that *Crataegus* bioactive compounds may exert a pleiotropic systemic effect—simultaneously modulating inflammation, oxidative damage, and cytokine signaling across multiple organ systems—rather than acting through organ-specific, independent pathways. However, this pleiotropic hypothesis has not itself been directly tested: no single study in the literature reviewed here has simultaneously assessed anti-inflammatory, cardioprotective, and neuroprotective endpoints within the same experimental model, so the assumption that a shared mechanism produces coordinated multi-organ benefit remains inferential rather than demonstrated.

Beyond these converging mechanisms, the literature reviewed in Section 4.2, Section 4.3 and Section 4.4 also contains unresolved tensions that a critical synthesis should acknowledge. First, the relative contribution of antioxidant versus direct electrophysiological mechanisms to the cardioprotective and antiarrhythmic effects of WS^®^1442 remains unclear: some studies emphasize redox-based mechanisms (reduced MDA, enhanced SOD/CAT), while others attribute the antiarrhythmic profile primarily to action-potential prolongation, and it is not established which mechanism predominates in vivo. Second, the hemostatic findings for WS^®^1442—elevated antithrombin III and reduced factor X activity—sit in tension with its promotion as a broadly cardioprotective agent, since these same properties raise a bleeding risk that could offset benefit in patients receiving anticoagulants. Third, within the neuroprotective literature, cholinergic mechanisms (cholinesterase inhibition by isolated triterpenoids) and antioxidant/anti-inflammatory mechanisms (reduced lipid peroxidation, restored antioxidant enzymes) are proposed as largely independent explanations for cognitive benefit, and no study has directly compared their relative contributions. These points indicate that the mechanistic picture, while broadly convergent at the level of NF-κB and oxidative stress, remains incompletely reconciled at the level of specific causal pathways.

### 4.5. Anticancer (Oncoprotective) Properties

Over the past decade, increasing evidence has highlighted the anticancer activity of *Crataegus* compounds, with polysaccharides and polyphenols, including flavonoids and proanthocyanidins, playing central roles. In preclinical models of colorectal cancer (CRC), hawthorn polysaccharides (HPS) administered at 50–200 mg/kg in an AOM/DSS-induced mouse model significantly reduced colonic tumor burden and inflammatory mediators, enhanced apoptosis and caspase-3 expression, and favorably modulated gut microbiota composition [96].

In HCT116 colorectal carcinoma cells, HPS inhibited proliferation, induced cell-cycle arrest in the S and G2/M phases, downregulated Cyclin A1/D1/E1 and CDK1/2 expression, and activated caspase-dependent apoptotic pathways involving caspases-3, -7, -8, and -9 [97]. Collectively, these findings are consistent with broader evidence indicating that hawthorn-derived extracts and bioactive constituents exert anticancer effects through multiple interconnected mechanisms, including inhibition of proliferation, induction of apoptosis and cell-cycle arrest, modulation of autophagy, and regulation of PI3K/Akt, Wnt/β-catenin, MAPK, NF-κB, and JAK/STAT signaling pathways [98].

Oligomeric proanthocyanidins from hawthorn (HPOE) demonstrate complementary anti-CRC activities: in HCT116 cells, they induce G2/M arrest via the p53–Cyclin B axis and trigger apoptosis through both mitochondrial (Caspase-9 → Caspase-3) and receptor-mediated (Caspase-8 → Caspase-3) pathways [99]. These compounds also suppress migration and invasion, as well as EMT markers (↓ N-cadherin, Vimentin, β-catenin; ↑ E-cadherin), by inactivating the Wnt/β-catenin pathway [100], indicating a strong anti-metastatic potential of HPOE.

The multitarget nature of *Crataegus* is supported by network and docking analyses: for *C. monogyna*, “anchor” molecules (luteolin, apigenin, quercetin, kaempferol, ursolic and oleanolic acids) have been identified as interacting with PPARG, MMP9, and key nodes such as AKT1, EGFR, and MAPK3, forming a basis for potential combination anticancer strategies [101].

Emerging evidence also suggests activity against hematological malignancies: an ethanol extract of “yellow” hawthorn berries reduced viability of CML (K562) and AML (MOLM-13) cells, inducing apoptosis and cell-cycle arrest (G0/G1 and G2/M). Although requiring further validation, these findings complement the broader picture of anticancer potential within the genus *Crataegus* [102].

Overall, contemporary preclinical evidence suggests that the anticancer potential of *Crataegus* is mediated through a combination of antiproliferative mechanisms (S/G2–M arrest), pro-apoptotic pathways (caspases-8/-9/-3), anti-metastatic effects (EMT and Wnt/β-catenin inhibition), and anti-inflammatory and microbiota-modulating actions [96,97,98,99,100,101,102].

It should be emphasized that all of the anticancer findings described above come from in vitro cell-line studies (HCT116, K562, MOLM-13) or short-term in vivo tumor models; no clinical or epidemiological data currently support an anticancer effect of *Crataegus* preparations in humans. While the reported mechanisms—apoptosis induction, cell-cycle arrest, and inhibition of epithelial–mesenchymal transition—are reasonably consistent across studies, the compound concentrations and routes of administration used in these preclinical models have not been validated against exposure levels achievable through oral consumption of *Crataegus* products.

Collectively, the available evidence demonstrates that *Crataegus* species exert a broad spectrum of pharmacological activities through multiple interconnected molecular mechanisms. Their biological effects are primarily attributed to polyphenols, flavonoids, proanthocyanidins, triterpenoids, and phenolic acids, which modulate oxidative stress, inflammatory signaling, apoptosis, autophagy, endothelial function, mitochondrial homeostasis, and cell-cycle regulation. These mechanisms contribute to the antioxidant, anti-inflammatory, cardioprotective, neuroprotective, and anticancer properties of *Crataegus*. However, the strength of evidence differs considerably among pharmacological domains. While cardiovascular benefits of the standardized extract WS^®^1442 are supported by clinical studies and meta-analyses, most antioxidant, neuroprotective, and anticancer mechanisms have been demonstrated predominantly in in vitro and animal models. Consequently, further well-designed clinical studies are required to validate the translational and therapeutic potential of *Crataegus* in human diseases. The principal bioactive compounds, experimental models, molecular targets, biological effects, and corresponding levels of evidence are summarized in Table 2.

## 5. Geroprotective Potential of *Crataegus*

**Figure 4:**
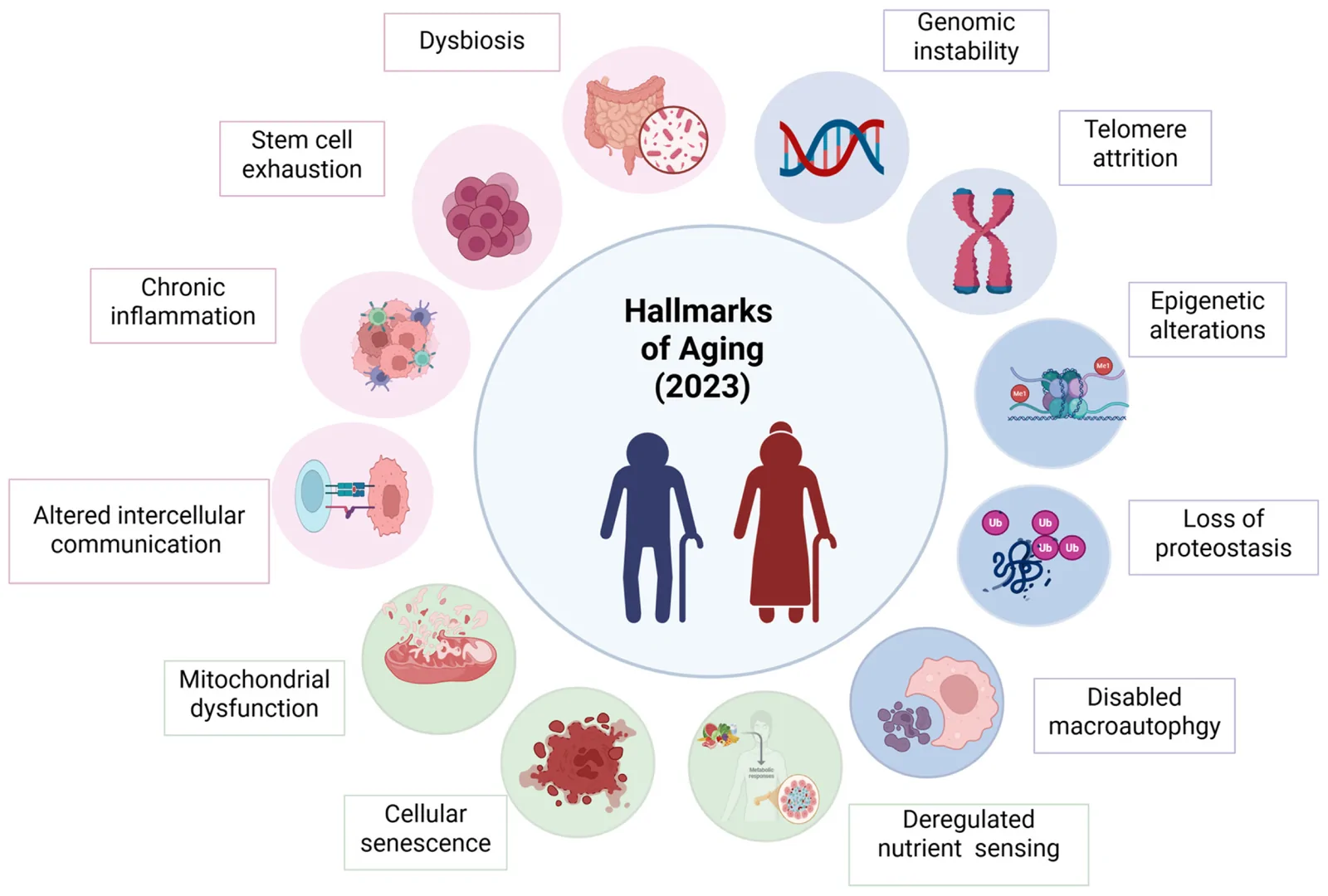
The twelve hallmarks of aging according to the updated 2023 framework. The figure summarizes the interconnected molecular and cellular mechanisms that drive biological aging, including genomic instability, telomere attrition, epigenetic alterations, loss of proteostasis, deregulated nutrient sensing, mitochondrial dysfunction, cellular senescence, stem cell exhaustion, altered intercellular communication, chronic inflammation, dysbiosis, and disabled macroautophagy. The illustration was created by the authors in BioRender (Created in BioRender. Kydyrbayeva, A. (2026) https://BioRender.com/5mqtnpf) based on the conceptual framework proposed by López-Otín et al. (2023) [2].

Although *Crataegus* species have traditionally been recognized for their cardioprotective, antioxidant, and anti-inflammatory properties, accumulating evidence indicates that their biological activity extends beyond conventional pharmacological effects and may modulate fundamental mechanisms underlying biological aging. The updated framework of the twelve hallmarks of aging proposed by López-Otín et al. (2023) [2] provides a comprehensive conceptual basis for understanding how natural products, including *Crataegus*, may influence the molecular and cellular processes that drive aging and age-related diseases (Figure 4).

Rather than acting through a single molecular target, accumulating evidence suggests that the diverse phytochemicals present in *Crataegus* may simultaneously modulate several interconnected aging-related pathways, supporting the concept of multitarget geroprotection. Against this conceptual background, recent studies have increasingly focused on the ability of *Crataegus* bioactive compounds to target multiple hallmarks of aging and thereby promote healthy aging and healthspan.

Recent reviews indicate that flavonoids commonly occurring in *Crataegus*, including quercetin, vitexin, rutin, and related polyphenolic compounds, can modulate aging-associated processes such as oxidative stress, cellular senescence, inflammatory signaling, and tissue dysfunction, supporting the potential relevance of flavonoid-rich *Crataegus* preparations to healthy-aging strategies [102,103].

Quercetin, one of the principal flavonoids of *Crataegus* species, has been reported to exert geroprotective effects through multi-target molecular and cellular mechanisms in isolated and cell-based models. In models of premature aging using human mesenchymal stem cells derived from Werner syndrome and Hutchinson—Gilford progeria, quercetin restored proliferative capacity and heterochromatin organization. It suppressed the expression of senescence markers, suggesting a potential role in genomic and epigenetic rejuvenation at the cellular level [104]. Its senolytic and senomorphic activity has also been confirmed in in vivo and cellular aging models. In *Simocephalus vetulus*, quercetin significantly increased both mean and maximum lifespan, activated AMPK-dependent energy metabolism, and directly regulated the expression of longevity-associated proteins Lamin A and Klotho [105]. In tendon stem/progenitor cell aging models, quercetin reduced SASP secretion, stabilized mitochondrial homeostasis, and suppressed the AKT/NF-κB/NLRP3 inflammatory axis, thereby restoring regenerative capacity in aged tissues. In addition, in aged mouse and human oocytes, quercetin decreased mitochondrial ROS levels, activated SIRT3-dependent deacetylation of SOD2, enhanced autophagy, and reduced apoptosis, leading to improved oocyte quality and reproductive potential during aging [106]. In H_2_O_2_-induced senescent BMSCs, quercetin reduced SA-β-gal activity, p21 expression, and SASP-associated inflammatory mediators, while restoring genomic and epigenomic stability and osteogenic differentiation. Mechanistically, these effects were associated with suppression of repetitive element activation, cytoplasmic dsRNA accumulation, and RIG-I-mediated innate immune signaling [107].

Epicatechin, a major flavanol present in *Crataegus* fruits and leaves, also demonstrates significant geroprotective activity through modulation of oxidative stress, energy metabolism, and tissue regeneration. Long-term dietary supplementation with epicatechin in aged C57BL/6 mice significantly increased survival, improved physical performance, delayed skeletal muscle degeneration, and restored age-altered extracellular matrix and nicotinamide-NAD^+^ metabolic pathways, indicating a robust systemic anti-aging effect in vivo [108]. Further evidence for the regenerative potential of flavonoids associated with *Crataegus* comes from studies combining quercetin and rutin with mesenchymal stem cell (MSC)-based therapies. In a cold-induced burn wound model, preconditioning of human umbilical cord-derived MSCs (hUC-MSCs) with quercetin or rutin enhanced cell migration and survival and improved wound healing. These effects were accompanied by downregulation of the pro-inflammatory cytokines IL-1β and IL-6, modulation of oxidative-stress-related genes (GPX7, PRDX7, and TXNRD2), increased expression of the regenerative and angiogenic factors VEGF and FGF, enhanced neovascularization, and improved tissue regeneration and re-epithelialization [109]. These findings suggest that bioactive flavonoids found in *Crataegus* may contribute not only to the modulation of cellular stress responses associated with aging but also to MSC-mediated tissue repair and regenerative processes.

Procyanidins, another vital polyphenol class occurring in *Crataegus*, have attracted particular attention because of their potential senotherapeutic properties. Procyanidin C1 (PCC1), a B-type trimeric procyanidin, exhibits both senomorphic and senolytic activity: at lower concentrations it suppresses the senescence-associated secretory phenotype (SASP), whereas at higher concentrations it selectively eliminates senescent cells through apoptosis associated with mitochondrial dysfunction and increased reactive oxygen species production. Intermittent administration of PCC1 to naturally aged mice improved physical function and prolonged post-treatment survival [110].

More recent single-cell transcriptomic evidence further demonstrated that long-term PCC1 treatment partially restored age-associated dysfunction of the hematopoietic and immune system, increased the proportions of B cells and hematopoietic stem cells, suppressed senescence- and inflammation-associated signatures, and promoted the elimination of senescent cell populations, particularly within granulocytic and myeloid compartments [111]. Rutin, another key flavonoid constituent of *Crataegus*, may also contribute to its potential geroprotective profile through antioxidant and anti-inflammatory mechanisms relevant to biological aging. Rutin exhibits potent free-radical-scavenging and lipid-peroxidation-inhibitory activities and can reinforce endogenous antioxidant defenses, thereby contributing to the maintenance of cellular redox homeostasis. Experimental evidence further indicates that rutin attenuates oxidative stress and inflammatory responses by modulating the NF-κB/MAPK signaling pathways, accompanied by reduced expression of inflammatory mediators, including TNF-α, IL-6, iNOS, and COX-2 [112]. Because these pathways are also implicated in age-associated chronic inflammation, these findings provide mechanistic support for the potential relevance of rutin to geroprotection. However, direct evidence from aging models remains limited.

Comparing the mechanisms described above for quercetin, epicatechin, procyanidin C1, and rutin reveals both convergence and compound-specific specialization. Antioxidant/ROS-scavenging activity is the most broadly shared mechanism, reported for quercetin (reduced mitochondrial ROS via SIRT3-SOD2 signaling), procyanidin C1 (reduced ROS production in senescent cells), and rutin (direct radical scavenging and enhanced SOD/catalase/GPx activity); epicatechin’s reported effects, in contrast, center on tissue-level metabolic and structural restoration (extracellular matrix, NAD^+^-dependent metabolism, skeletal muscle) rather than direct radical scavenging. NF-κB suppression is shared by quercetin (via the AKT/NF-κB/NLRP3 axis) and rutin (via NF-κB/MAPK signaling), linking both compounds to the same inflammaging pathway discussed in Section 4.2.

A more consequential distinction concerns senescent-cell clearance versus SASP suppression: procyanidin C1 is the only one of the four compounds described here as directly senolytic, selectively inducing apoptosis in senescent cells at higher concentrations, whereas quercetin and rutin are more accurately characterized as senomorphic, suppressing SASP secretion and pro-inflammatory signaling without necessarily eliminating the senescent cells themselves. Epigenetic and chromatin-level effects (heterochromatin reorganization, suppression of RIG-I-mediated repetitive-element sensing) have so far been reported only for quercetin, and tissue-specific regenerative effects (muscle, extracellular matrix, wound re-epithelialization) only for epicatechin, indicating that these two compounds may contribute distinct, non-overlapping benefits rather than acting solely through the shared antioxidant/NF-κB axis. Overall, the four compounds appear to converge on a common antioxidant and anti-inflammatory core—most clearly linking quercetin and rutin—while diverging in their capacity for direct senescent-cell elimination (procyanidin C1), epigenetic remodeling (quercetin), and tissue regeneration (epicatechin), a pattern consistent with synergistic, complementary action within the intact *Crataegus* extract rather than redundant activity of a single dominant compound.

**Figure 5:**
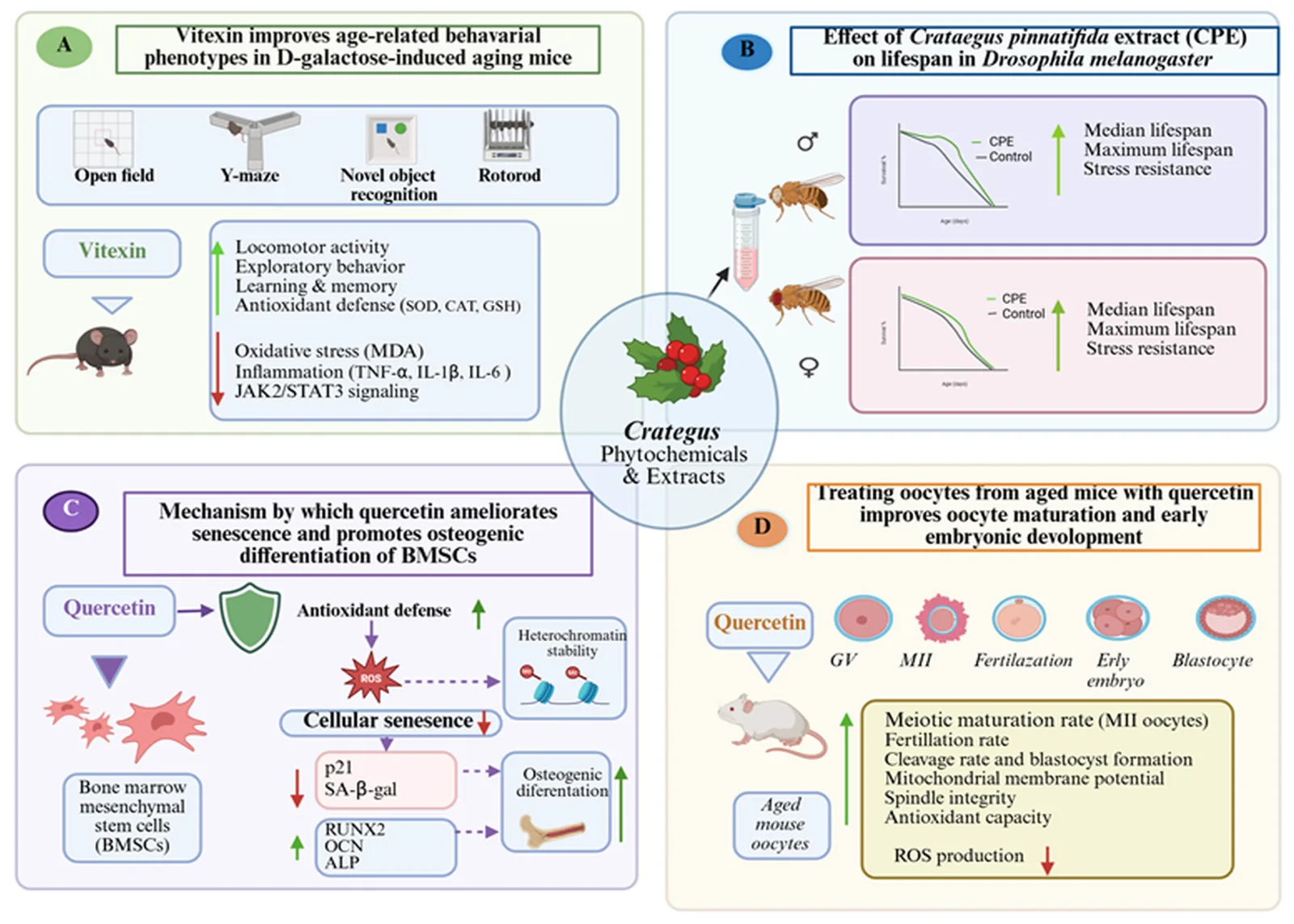
Overview of the anti-aging effects of major *Crataegus* phytochemicals and extracts. (**A**) Vitexin attenuates D-galactose-induced aging by improving behavioral performance, enhancing antioxidant defense, and suppressing oxidative stress, inflammation, and JAK2/STAT3 signaling, based on the findings of Han et al. [113]. (**B**) *Crataegus pinnatifida* extract (CPE) prolongs lifespan and enhances stress resistance in *Drosophila melanogaster*, based on the findings of Yu et al. [114]. (**C**) Quercetin alleviates bone marrow mesenchymal stem cell (BMSC) senescence by enhancing antioxidant defense, reducing reactive oxygen species (ROS), preserving heterochromatin stability, decreasing cellular senescence markers (p21 and SA-β-gal), and promoting osteogenic differentiation, based on the findings of Sun et al. [108]. (**D**) Quercetin improves the quality of aged mouse oocytes by enhancing meiotic maturation, fertilization, early embryonic development, mitochondrial function, and antioxidant capacity while reducing intracellular ROS, based on the findings of Cao et al. [107]. The figure was created by the authors based on published experimental findings from references [107,108,113,114]. ↑ indicates an increase or activation; ↓ indicates a decrease, inhibition, or downregulation.

Taken together, these findings indicate that the geroprotective activity of *Crataegus* is supported by evidence obtained from diverse experimental models and involves multiple complementary molecular mechanisms. Representative studies illustrating the anti-aging effects of major *Crataegus* phytochemicals and extracts are summarized in Figure 5.

Additional phenolic constituents of *Crataegus*, including caffeic acid and related hydroxycinnamic acids, are plausible contributors to its indirect geroprotective activity, consistent with the antioxidant and anti-inflammatory properties described for these compounds in recent phytochemical and anti-aging studies of *Crataegus* [61,104]. Attenuation of oxidative stress, modulation of inflammatory signaling pathways, particularly NF-κB, neuroprotection, and suppression of age-related inflammation and DNA damage have been proposed as potential mechanisms through which these phenolic acids may contribute to healthy aging. However, these mechanisms have not yet been experimentally demonstrated specifically for caffeic acid derived from *Crataegus*, and therefore their contribution to the geroprotective effects of the whole plant remains to be clarified. Collectively, the synergistic action of epicatechin, procyanidins, rutin, phenolic acids, and other bioactive constituents likely underlies the multitarget geroprotective potential of *Crataegus*, operating through antioxidant defense, attenuation of cellular senescence, regulation of autophagy, mitochondrial protection, suppression of SASP, and maintenance of immune homeostasis [61,104].

**Figure 6:**
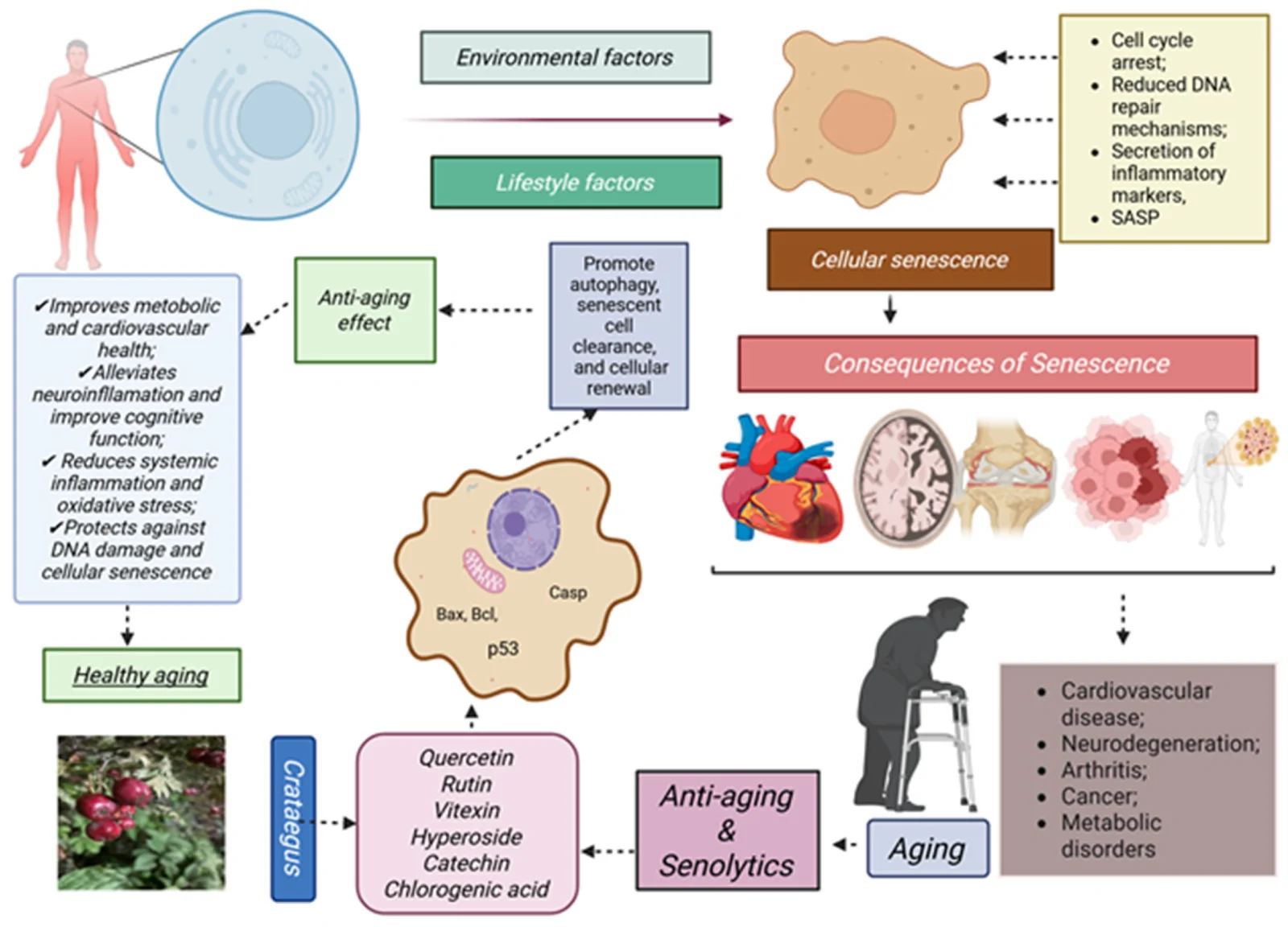
Proposed anti-aging and senolytic mechanisms of *Crataegus* bioactive compounds. Major *Crataegus* phytochemicals, including quercetin, rutin, vitexin, hyperoside, catechin, and chlorogenic acid, may counteract cellular senescence through multiple complementary mechanisms, including the promotion of autophagy, enhancement of cellular renewal, suppression of the senescence-associated secretory phenotype (SASP), reduction in oxidative stress, improvement of DNA repair capacity, and modulation of apoptosis in senescent cells. Collectively, these mechanisms may contribute to the prevention of age-related diseases, attenuation of tissue dysfunction, and maintenance of cellular homeostasis, thereby promoting healthy aging. The figure was created by the authors based on published experimental findings and generated using BioRender.com (Created in BioRender. Kydyrbayeva, A. (2026) https://BioRender.com/nmekgnn).

Collectively, the available evidence suggests that the diverse phytochemicals of *Crataegus* act synergistically to exert multitarget geroprotective effects rather than acting through a single molecular mechanism. Epicatechin, procyanidins, rutin, phenolic acids, and other bioactive constituents collectively contribute to the attenuation of oxidative stress, suppression of chronic inflammation and cellular senescence, regulation of autophagy, preservation of mitochondrial function, modulation of apoptosis, inhibition of the senescence-associated secretory phenotype (SASP), and maintenance of cellular homeostasis. Through these complementary mechanisms, *Crataegus* may help delay the onset of age-related functional decline and promote healthy aging. The proposed interactions between the major bioactive compounds and the principal molecular pathways involved in geroprotection are summarized in Figure 6 [61,104].

**Targets and Mechanisms**

The key mechanistic directions relevant to geroprotection include:

1. Redox homeostasis and Nrf2/ARE activation. In ovariectomized rats, fruit extract of *Crataegus pinnatifida* upregulated Nrf2/HO-1/GPx expression and improved the lipid profile while reducing hepatic MDA—an integral marker of lipid peroxidation [115]. These findings indicate restoration of antioxidant defenses under estrogen-deficiency–induced oxidative stress. A comparable redox-based mechanism has been reported for other plant-derived polysaccharides: in a D-galactose-induced heart aging model in mice, *Polygonatum sibiricum* polysaccharides reduced myocardial reactive oxygen species and malondialdehyde while increasing superoxide dismutase activity, supporting the broader concept that phytochemical antioxidants can attenuate age-related cardiac oxidative damage across different plant sources [116].
2. Inhibition of SASP and pro-inflammatory cascades. Vitexin—a characteristic *Crataegus* flavonoid—reduced SASP and suppressed JAK2/STAT3 signaling in in vivo and in vitro models of induced senescence, demonstrating a clear anti-senescent effect [117]. This mechanistically aligns with review data highlighting the role of flavonoids in attenuating pro-inflammatory aging phenotypes [103].
3. IIS/PI3K—Akt, TOR/autophagy pathways, and stress resistance. In *Drosophila*, *C. pinnatifida* extract extended lifespan in both sexes and modulated TOR/4EBP markers, autophagy (Atg5/Atg8a), Notch/telomere regulation, and the JNK stress response, without negatively affecting fertility or feeding behavior [113]. Similarly, in *C. elegans*, fruit extract increased lifespan by ~28% and enhanced resistance to oxidative and thermal stress via IIS signaling [114].
4. Endothelial senescence as a systemic therapeutic target. Endothelial senescence represents an early and pathologically significant component of vascular aging; the anti-inflammatory and antioxidant properties of *Crataegus* are well-positioned to counteract this node [118]. The available evidence on the geroprotective effects of *Crataegus*, including the experimental models, bioactive compounds or extracts, molecular targets, and reported effects, is summarized in Table 3.

### 5.1. Composition of Active Constituents and Formulations

*Crataegus* fruits are rich in phenolic acids (including gallic and caffeic acids), flavonoids (quercetin, vitexin, vitexin-2″-O-rhamnoside, hyperoside, and rutin), proanthocyanidins, and pectins; their phytochemical profile varies depending on species, geographical origin, and extraction conditions [33,36,47,121]. This phytochemical diversity underlies the multitarget pharmacological effects of *Crataegus*, including antioxidant, anti-inflammatory, neuroprotective, and cardioprotective activities, and supports its potential application as a nutraceutical and cosmeceutical resource [103,121].

*Crataegus* fruits are rich in phenolic acids (gallic, caffeic), flavonoids (quercetin, vitexin/vitexin-2″-O-rhamnoside, hyperoside, rutin), proanthocyanidins, and pectins; the phytochemical profile varies depending on species/region and extraction methodology [121]. This diversity underlies both the plant’s multitarget pharmacodynamics (antioxidant, anti-inflammatory, neuroprotective, cardioprotective) and its potential for nutraceutical and cosmeceutical applications [103].

### 5.2. Limitations and Knowledge Gaps

Despite convincing in vitro findings, rodent data, and results from “small” aging models, large-scale clinical studies evaluating geroprotective outcomes (telomeric/epigenetic clocks, functional domains of healthspan) for *Crataegus* remain scarce and predominantly focused on dermatological parameters [119,120]. Confirmation of systemic geroprotection in humans using validated biomarkers and hard clinical endpoints is urgently needed.

Specifically, validated aging biomarkers that could substantiate translational claims for *Crataegus* include epigenetic clocks (e.g., Horvath or PhenoAge clocks), mitochondrial DNA copy number, and composite inflammatory indices; none of these have yet been measured in a clinical trial of *Crataegus* preparations. Well-designed studies would additionally require standardized, chemically characterized extracts, dose-ranging protocols, and follow-up periods long enough to capture changes in slow biological processes such as epigenetic aging, since the short observation windows used in most cited animal studies cannot address whether effects persist or scale to human lifespans.

A further limitation concerns the model organisms used to demonstrate lifespan extension (*Drosophila*, *Caenorhabditis elegans*, *rodents*): differences in metabolic rate, genome organization, and absolute compound exposure relative to body mass mean that percentage lifespan gains observed in these organisms cannot be assumed to translate proportionally to humans. Similarly, as noted throughout Section 5, much of the mechanistic evidence for individual flavonoids (quercetin, epicatechin, procyanidin C1) was generated using purified compounds rather than whole *Crataegus* extracts, so the relationship between compound-level activity and the geroprotective potential of the intact plant extract—which may involve synergistic or antagonistic interactions among its many constituents—remains to be established experimentally.

### 5.3. Safety, Pharmacokinetics, and Translational Considerations

Clinical translation of the geroprotective effects described above requires careful attention to safety and pharmacokinetics, an aspect that has received comparatively little attention in the *Crataegus* geroprotection literature. Standardized extracts such as WS^®^1442 have documented dosing regimens and a favorable safety profile in chronic heart failure patients, but most other *Crataegus* preparations discussed in this review lack comparable pharmacokinetic characterization. Oral bioavailability of the principal flavonoids (quercetin, rutin, hyperoside) and procyanidins is generally low, owing to extensive first-pass metabolism and limited intestinal absorption of high-molecular-weight polyphenols, raising the question of whether concentrations sufficient to reproduce the antioxidant, anti-inflammatory, and senolytic effects observed in vitro are achievable through standard oral dosing in humans.

Regarding safety, *Crataegus* preparations are generally well tolerated, but specific interactions warrant caution. This is corroborated by the European Union herbal monograph on *Crataegus* spp., folium cum flore, compiled by the Committee on Herbal Medicinal Products (HMPC) of the European Medicines Agency, which reports no known undesirable effects or drug interactions for well-established-use hawthorn leaf-and-flower preparations, while advising caution in children, during pregnancy and lactation, and recommending medical consultation if cardiac symptoms persist or worsen [122]. As noted in Section 4.3, WS^®^1442 elevates antithrombin III levels and reduces factor X activity, indicating a potential bleeding risk in patients receiving anticoagulant or antiplatelet therapy. Because *Crataegus* extracts are frequently used by patients with cardiovascular disease—the same population most likely to be prescribed digoxin or other cardiac glycosides—potential pharmacodynamic interactions with these agents deserve dedicated study; to date, this interaction has not been systematically investigated for *Crataegus*, in contrast to the more thoroughly characterized toxicology and herb-drug interaction profiles established through systematic reviews of other medicinal botanicals [123].

More broadly, standardization of *Crataegus* phytopreparations—in terms of extraction method, marker-compound content, and dosage form—remains inconsistent across the studies reviewed here (Table 1), complicating cross-study comparison of both efficacy and safety data. Future translational research on the geroprotective potential of Crataegus should therefore prioritize standardized extract characterization, formal pharmacokinetic and drug-interaction studies, and dose-ranging safety trials before large-scale clinical evaluation of aging-related endpoints is undertaken.

### 5.4. Synthesis and Future Directions

The accumulated evidence shows that *Crataegus* possesses multifactorial geroprotective potential mediated through enhancement of Nrf2-dependent cytoprotection, attenuation of SASP and inflammatory cascades (including JAK2/STAT3), modulation of IIS/PI3K–Akt and TOR/autophagy pathways, and a possible influence on endothelial senescence. These mechanisms align with experimental and clinical outcomes—lifespan extension in model organisms, increased stress resilience, and early signs of improved “skin age’’ in humans.

Promising future directions include multicenter randomized controlled trials assessing epigenetic clocks and systemic biomarkers of aging, together with standardized extraction and dosing protocols.

### 5.5. Prospects for the Use of Crataegus in Gerontology and Biomedicine

Among the most promising sources of geroprotective agents are medicinal plants, whose long-standing use is well documented in traditional medicine. Because plant extracts represent complex mixtures of bioactive molecules capable of simultaneously modulating hundreds of human molecular targets [124], their polypharmacology is increasingly regarded as a key factor contributing to pronounced geroprotective potential, often offering advantages over single-target agents of conventional pharmacotherapy [125]. This multitarget capacity is illustrated directly within this review by two independent network-pharmacology analyses of *Crataegus* constituents, which identified overlapping and complementary molecular targets—PTGS2/COX-2, EGFR, and MMP2/9 in a coronary artery disease model [82], and PPARG, MMP9, AKT1, and MAPK3 in a breast cancer model [101]—spanning cardiovascular and oncological contexts and reinforcing the concept that a shared pool of *Crataegus* flavonoids converges on a limited set of druggable nodes across disease states.

Beyond the antioxidant, anti-inflammatory, and cardioprotective mechanisms already detailed in Section 4, procyanidins in particular have also been reported to show antibacterial and antiviral activity [71], broadening the potential relevance of *Crataegus* polypharmacology beyond the mechanisms discussed above. It should be noted, however, that antioxidants are not always capable of fully suppressing excessive free-radical formation at late disease stages, underscoring the importance of early, preventive use of plant-derived compounds such as these rather than reliance on them once age-related pathology is advanced [125].

### 5.6. Distinguishing Preclinical and Clinical Evidence

An essential translational consideration for *Crataegus* geroprotection research is the clear separation of evidence by level of validation. At present, the findings summarized in this review derive from three tiers of evidence with markedly different translational weight: (i) in vitro and cell-based studies (chemical antioxidant assays, cell-culture senescence models), which establish mechanistic plausibility but cannot demonstrate efficacy in an intact organism; (ii) small-scale in vivo studies in model organisms and rodents, which demonstrate biological activity under controlled experimental conditions but often use doses, routes of administration, or genetic backgrounds that do not directly correspond to human physiology; and (iii) human clinical studies, which remain the only tier capable of establishing efficacy and safety in the target population, but which for *Crataegus* are largely restricted to cardiovascular endpoints (e.g., the WS^®^1442 trials) rather than aging-related endpoints.

Explicitly distinguishing between these tiers is essential to avoid overstating the current level of evidence. The cardioprotective, antihypertensive, and antiarrhythmic effects of standardized *Crataegus* extracts have direct clinical support from randomized controlled trials and large cohort studies (Section 4.3). In contrast, the specific geroprotective mechanisms discussed in Section 5—senescent cell clearance, epigenetic rejuvenation, lifespan extension in model organisms—remain at the preclinical stage: no clinical trial to date has assessed validated aging biomarkers or hard clinical endpoints in humans following *Crataegus* administration. Readers and future investigators should therefore interpret the geroprotective potential of *Crataegus* as a well-supported preclinical hypothesis, built on an established clinical safety and cardiovascular efficacy base, rather than as a clinically validated anti-aging intervention.

## 6. Conclusions

Current experimental and limited clinical evidence indicates that *Crataegus* species, including the endemic *C. almaatensis*, possess notable geroprotective potential through their rich phytochemical profile and multi-target mechanisms of action. Reported bioactive constituents—flavonoids, procyanidins, phenolic acids, and triterpenoids—modulate several hallmarks of aging by reducing oxidative stress via Nrf2/ARE signaling, suppressing SASP and inflammatory pathways (NF-κB, JAK2/STAT3), and regulating IIS–PI3K/Akt and mTOR/autophagy signaling. These effects contribute to improved endothelial function. Preclinical studies in *Drosophila*, *C. elegans*, rats, and mice have demonstrated lifespan extension, increased stress resistance, and neuro- and cardioprotective effects.

However, most of this evidence remains at the cellular or animal-model level, and its translation to whole-organism, human-relevant outcomes is not yet established. Key gaps include the absence of validated aging biomarkers (e.g., epigenetic clocks, mtDNA copy number, inflammatory age index) measured in *Crataegus* studies; uncertainty about which route and form of administration most reliably achieves bioactive concentrations in target organs; and a scarcity of well-designed clinical trials with adequate sample size, duration, and extract standardization. For *C. almaatensis* specifically, organ-specific in vivo studies (liver, heart, kidney, brain) are planned but have not yet been completed.

Overall, *C. almaatensis* represent promising candidate for the development of nutraceutical and pharmaceutical geroprotectors, particularly as part of combined anti-aging strategies. Future work should prioritize identification of marker compounds, standardized extract preparations, and randomized clinical trials using validated biomarkers to confirm whether the geroprotective effects observed preclinically extend to humans.

## Figures and Tables

**Table 2:** Principal bioactive preparations, experimental models, molecular targets, and pharmacological effects of *Crataegus* species.

Pharmacological Domain	Compound or Extract	Experimental Model	Principal Signaling Pathway or Molecular Target	Main Biological Effect	Evidence Level	Ref.
**Antioxidant**	Alcoholic extract of *C. monogyna* fruits, rich in (−)-epicatechin, catechin, and procyanidins B1/B2	DPPH•, ABTS•+ and reducing-power assays	Direct radical scavenging, electron/proton transfer and reducing activity; no intracellular pathway assessed	Neutralization of free radicals and increased reducing capacity	Cell-free biochemical assays	[65]
**Antioxidant**	Aqueous and alcoholic extracts of *C. monogyna* flowers and leaves	DPPH•, ABTS•+, reducing-power and Fe^2+^-chelating assays	Direct radical scavenging and metal-ion chelation; no intracellular pathway assessed	Reduced free-radical availability and Fe^2+^-dependent oxidative reactions	Cell-free biochemical assays	[66]
**Antioxidant**	70% ethanolic extract of *C. laevigata* berries	LPS-stimulated HaCaT keratinocytes	↓ MAPK/AP-1, ↓ NF-κB and ↓ NFAT activation	↓ Intracellular ROS and attenuation of oxidative–inflammatory signaling	In vitro	[67]
**Antioxidant**	Polyphenol-rich *C. oxyacantha* extract	CCl_4_-induced liver fibrosis in rats	↓ TGF-β1, ↓ NF-κB and ↓ α-SMA; ↑ SOD	↓ MDA and protein carbonyls; reduced collagen deposition and restoration of liver histoarchitecture	In vivo	[68]
**Antioxidant**	Aqueous *C. oxyacantha* extract	Deltamethrin- and chlorpyrifos-induced neurotoxicity in rats	↑ SOD, ↑ CAT, ↑ GPx and ↑ GSH; ↓ lipid peroxidation	Protection against oxidative brain injury, DNA fragmentation and neurobehavioral impairment	In vivo	[69]
**Anti-inflammatory**	70% ethanolic extract of *C. laevigata* berries	LPS-stimulated HaCaT keratinocytes	↓ MAPK/AP-1, ↓ NF-κB and ↓ NFAT	↓ ROS, TARC/CCL17, MDC/CCL22, RANTES/CCL5 and IL-8	In vitro	[67]
**Anti-inflammatory**	Triterpenoids isolated from *C. pinnatifida* leaves	LPS-stimulated RAW 264.7 macrophages; molecular docking and enzymatic assays	Direct SRC inhibition followed by suppression of STAT3 signaling	↓ NO, iNOS, COX-2, TNF-α, IL-1β and IL-6	In vitro and in silico	[75]
**Anti-inflammatory**	Ursolic acid	Inflammatory cellular and experimental models summarized in the cited studies	↓ NF-κB nuclear translocation; ↓ COX-2 and ↓ iNOS	Reduced production of pro-inflammatory cytokines and attenuation of inflammation-associated cellular injury	Preclinical evidence	[76,77]
**Anti-inflammatory**	Hydroethanolic extract of *C. monogyna* leaves and stems	Carrageenan-induced paw edema and acetaminophen-induced hepatotoxicity in rats	Molecular pathway not directly assessed	Dose-dependent anti-edematous activity and hepatoprotection; approximately 99% edema inhibition at 6 h at 1000 mg/kg	In vivo	[45]
**Anti-inflammatory**	Phenolic seed fractions from six *Crataegus* species, rich in B-type procyanidins and flavan-3-ols	Enzyme-inhibition assays	Inhibition of LOX, COX-2 and XO	Reduced leukotriene-associated and cyclooxygenase-dependent inflammatory activity	Cell-free biochemical assays	[48]
**Cardioprotective**	WS^®^1442 standardized leaf-and-flower extract	Retrospective cohort of 9100 patients; 5-year follow-up	Molecular pathway not directly assessed in the cohort	Lower incidence of atrial fibrillation/flutter, tachycardia and other arrhythmias; HR for atrial fibrillation = 0.71	Clinical, retrospective cohort	[81]
**Cardioprotective**	Quercetin, isorhamnetin and kaempferol from *Crataegus* leaves	Network pharmacology analysis of coronary artery disease	PTGS2/COX-2, EGFR, MMP2, MMP9 and inflammation-related targets	Predicted coordinated regulation of inflammatory, endothelial and extracellular-matrix processes	In silico	[82]
**Cardioprotective**	WS^®^1442 and its oligomeric procyanidin fraction	Embryonic stem cells and cardiac progenitor cells	TGF-β/BMP and FGF signaling; ↑ BDNF and retinoic-acid-associated signaling	Promotion of cardiomyogenesis and angiogenesis	In vitro	[84]
**Cardioprotective**	*Crataegus* preparations evaluated in randomized placebo-controlled trials	Patients with hypertension; pooled randomized trials	Molecular pathway not directly assessed	Reduction in systolic blood pressure by approximately 6.6 mmHg after 2–6 months	Meta-analysis of clinical trials	[86]
**Cardioprotective**	WS^®^1442	Myocardial ischemia–reperfusion injury in rats	↓ NF-κB; ↑ SOD and CAT; activation of eNOS/NO signaling; ↓ Ca^2+^ overload	↓ Infarct size, ventricular arrhythmias, inflammatory mediators and mortality; improved post-ischemic contractility	In vivo	[87]
**Cardioprotective/safety**	WS^®^1442	Hemostatic assessment in rats	↑ Antithrombin III and ↓ factor X activity	Anticoagulant-related hemostatic modulation; potential bleeding concern when combined with anticoagulants or antiplatelet agents	In vivo safety evidence	[89]
**Neuroprotective**	*Crataegus* extract, 100–1000 mg/kg	Streptozotocin-induced diabetic rats	Specific molecular pathway not directly assessed; metabolic modulation reported	Improved passive-avoidance learning and reduced glucose and cholesterol levels	In vivo	[94]
**Neuroprotective**	Aqueous *C. oxyacantha* extract	Deltamethrin- and chlorpyrifos-induced neurotoxicity in rats	↑ SOD, CAT, GPx and GSH; ↓ lipid peroxidation	Prevention of behavioral impairment, oxidative brain damage, DNA fragmentation and histopathological injury	In vivo	[69]
**Neuroprotective**	Nine isolated constituents from *C. oxyacantha*, including lupeol, betulin, betulinic acid, oleanolic acid and chrysin	AChE/BChE enzyme-inhibition assays with predicted blood–brain barrier permeability	Inhibition of AChE and BChE	Cholinesterase inhibition; lowest reported AChE IC_50_ approximately 5.22 μM	In vitro *and* in silico	[91]
**Neuroprotective**	Total flavonoids from hawthorn leaves (TFHL), 5–20 mg/kg	Experimental spinal cord injury in rats	↓ Bax and cleaved caspase-3; ↑ Bcl-2	Reduced neuronal apoptosis and lesion size; improved BBB locomotor score	In vivo	[92]
**Neuroprotective**	Total flavonoids from hawthorn leaves	Spinal motor neuron–astrocyte co-culture and spinal cord injury models	Activation of autophagy-associated proteins and neurotrophic signaling	Enhanced axonal regeneration, neurotrophic-factor production and neurological recovery	In vitro and in vivo	[92]
**Neuroprotective**	*Crataegus* supplementation combined with physical training	Trimethyltin-induced neurotoxicity in rats	↑ BDNF/TrkB; ↓ AChE activity and amyloid burden	Improved spatial learning and reduced neuronal necrosis, astrocytosis and microgliosis	In vivo	[93]
**Anticancer**	Water-soluble hawthorn polysaccharides, 50–200 mg/kg	Experimental colorectal tumor model; 9-week administration	↑ Caspase-3; modulation of inflammatory responses and gut microbiota	Reduced tumor number and inflammatory markers; increased apoptosis and favorable microbiota changes	In vivo	[96]
**Anticancer**	Water-soluble hawthorn polysaccharides	HCT116 human colorectal carcinoma cells	↓ Cyclins A1/D1/E1 and CDK1/2; activation of caspases-3/7/8/9	S/G2–M cell-cycle arrest and induction of apoptosis	In vitro	[97]
**Anticancer**	Hawthorn polysaccharides	Colorectal cancer cell models	MAPK-associated apoptotic signaling and caspase-3 activation	Reduced proliferation and increased apoptotic cell death	In vitro	[98]
**Anticancer**	Hawthorn oligomeric proanthocyanidins	HCT116 human colorectal carcinoma cells	p53–Cyclin B axis; caspase-9 → caspase-3 and caspase-8 → caspase-3 pathways	G2/M arrest and activation of intrinsic and extrinsic apoptosis	In vitro	[99]
**Anticancer**	Hawthorn oligomeric proanthocyanidins	Colorectal cancer migration and invasion models	Inhibition of Wnt/β-catenin signaling; ↓ N-cadherin, vimentin and β-catenin; ↑ E-cadherin	Suppression of epithelial–mesenchymal transition, migration and invasion	In vitro	[100]
**Anticancer**	Luteolin, apigenin, quercetin, kaempferol, ursolic acid and oleanolic acid from *C. monogyna*	Network pharmacology and molecular docking	PPARG, MMP9, AKT1, EGFR and MAPK3	Predicted multitarget anticancer and anti-invasive activity	In silico	[101]
**Anticancer**	Ethanolic extract of yellow hawthorn berries	K562 chronic myeloid leukemia and MOLM-13 acute myeloid leukemia cells	Cell-cycle and apoptosis-related mechanisms; individual molecular targets not fully characterized	Reduced cell viability, induced apoptosis and caused G0/G1 or G2/M arrest	In vitro	[102]

Note: Bold indicates pharmacological domains. ↑, increase or activation; ↓, decrease, inhibition, or downreg-ulation.

**Table 3:** Geroprotective Effects of *Crataegus*.

Model	Compound/Extract	Molecular Target	Effect	Reference
Rats (ovariectomy)	*C. pinnatifida* fruit extract	↑ Nrf2/HO-1/GPx; ↓ MDA; improved lipid profile	Restoration of antioxidant defense under estrogen-deficiency–induced stress	[115]
In vivo/in vitro (induced senescence)	Vitexin	↓ SASP; inhibition of JAK2/STAT3	Anti-senescent effect	[117]
*Drosophila melanogaster*	*C. pinnatifida* extract	Modulation of TOR/4EBP; autophagy (Atg5/Atg8a); Notch/telomere regulation; JNK response	Lifespan extension without impairing fertility/feeding	[113]
*Caenorhabditis elegans*	*Crataegus* fruit extract	IIS pathway involvement	~28% lifespan extension; ↑ resistance to oxidative/thermal stress	[114]
Humans (healthy volunteers)	Combination of *C. pinnatifida* + *Panax ginseng* (topical/oral)	↓ MMP-1; ↑ procollagen I (in vitro); dermatological improvements (in vivo)	Skin anti-aging effects	[119]
Humans (6-month randomized trial)	*Crataegus* fruit powder	Association with TERT polymorphisms; correlation with telomere length	↑ Skin hydration; link with biomarkers of biological aging	[120]

Note: ↑ indicates an increase or activation; ↓ indicates a decrease, inhibition, or downregulation.

## Data Availability

No new data were created or analyzed in this study. Data sharing is not applicable to this article.

## Abbreviations

The following abbreviations are used in this manuscript:

AMPK—AMP	activated protein kinase
ARE	antioxidant response element
ATP	adenosine triphosphate
BDNF	brain-derived neurotrophic factor
BMSCs	bone marrow mesenchymal stem cells
CPE	*Crataegus pinnatifida* extract
D-gal	D-galactose
DNA	deoxyribonucleic acid
GPx	glutathione peroxidase
HO-1	heme oxygenase-1
HPLC-DAD	high-performance liquid chromatography with diode-array detection
H_2_O_2_	hydrogen peroxide
IIS	insulin/insulin-like signaling
IL	interleukin
iNOS	inducible nitric oxide synthase
JAK2	Janus kinase 2
MAPK	mitogen-activated protein kinase
MeJA	methyl jasmonate
MMP	matrix metalloproteinase
mTOR	mechanistic target of rapamycin
NF-κB	nuclear factor kappa B
Nrf2	nuclear factor erythroid 2-related factor 2
OPCs	oligomeric proanthocyanidins
PCC1	procyanidin C1
PI3K	phosphoinositide 3-kinase
ROS	reactive oxygen species
SASP	senescence-associated secretory phenotype
SIRT3	sirtuin 3
SRC	proto-oncogene kinase
STAT3	signal transducer and activator of transcription 3
TERT	telomerase reverse transcriptase
TFC	total flavonoid content
TPC	total phenolic content
TNF-α	tumor necrosis factor alpha
TOR	target of rapamycin
